# Dynamics of tumor-associated macrophages in a quantitative systems pharmacology model of immunotherapy in triple-negative breast cancer

**DOI:** 10.1016/j.isci.2022.104702

**Published:** 2022-06-30

**Authors:** Hanwen Wang, Chen Zhao, Cesar A. Santa-Maria, Leisha A. Emens, Aleksander S. Popel

**Affiliations:** 1 Department of Biomedical Engineering, Johns Hopkins University School of Medicine, Baltimore, MD 21205, USA; 2 Department of Oncology, the Sidney Kimmel Cancer Center, Johns Hopkins University School of Medicine, Baltimore, MD21205, USA; 3 University of Pittsburgh Medical Center, Hillman Cancer Center, Pittsburgh, PA, USA; 4 School of Pharmacy, Nanjing Medical University, Nanjing, Jiangsu211166, China

**Keywords:** Pharmacology, Immunology, Biophysics, Cancer

## Abstract

Quantitative systems pharmacology (QSP) modeling is an emerging mechanistic computational approach that couples drug pharmacokinetics/pharmacodynamics and the course of disease progression. It has begun to play important roles in drug development for complex diseases such as cancer, including triple-negative breast cancer (TNBC). The combination of the anti-PD-L1 antibody atezolizumab and nab-paclitaxel has shown clinical activity in advanced TNBC with PD-L1-positive tumor-infiltrating immune cells. As tumor-associated macrophages (TAMs) serve as major contributors to the immuno-suppressive tumor microenvironment, we incorporated the dynamics of TAMs into our previously published QSP model to investigate their impact on cancer treatment. We show that through proper calibration, the model captures the macrophage heterogeneity in the tumor microenvironment while maintaining its predictive power of the trial results at the population level. Despite its high mechanistic complexity, the modularized QSP platform can be readily reproduced, expanded for new species of interest, and applied in clinical trial simulation.

## Highlights

• A mechanistic model of quantitative systems pharmacology in immuno-oncology
• Dynamics of tumor-associated macrophages are integrated into our previous work
• Conducting in silico clinical trials to predict clinical response to cancer therapy

## Graphical abstract

**Figure:**
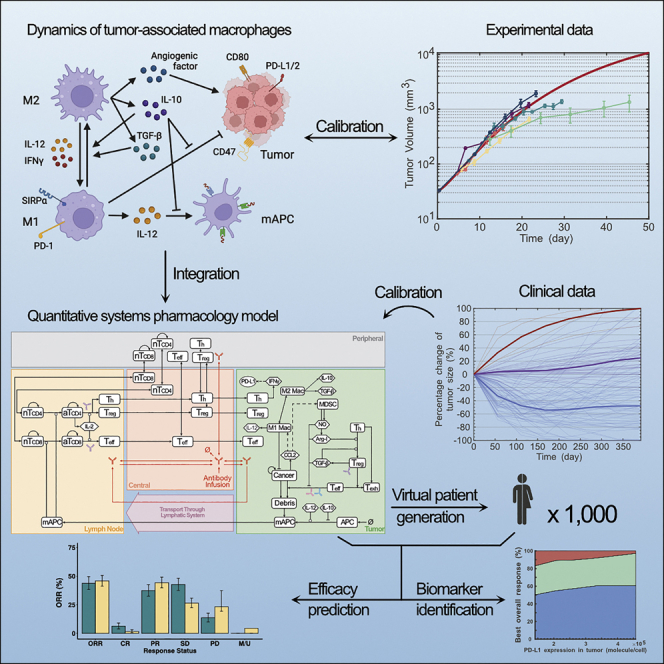

## Introduction

Breast cancer is the most commonly diagnosed cancer with over two million new cases worldwide annually (Ferlay et al., 2021). Triple-negative breast cancer (TNBC), which is defined by its lack of estrogen receptor, progesterone receptor, and human epidermal growth factor receptor-2 expressions, accounts for around 15% of total breast cancer cases. TNBC is considered an aggressive phenotype of breast cancer, and while patients can respond well to chemo-immunotherapy, those with residual disease have high rates of recurrence with approximately one-third developing recurrent disease within three years (Hensing et al., 2020; Schmid et al., 2020). Once metastatic, the disease is incurable and overall survival is substantially lower compared to other types of breast cancer (Lee et al., 2020; Lin et al., 2012). Although chemotherapy remains the mainstay treatment for patients with metastatic TNBC, targeted therapies such as sacituzumab-govitecan have recently been developed (Bardia et al., 2021; Malhotra and Emens, 2020). Biomarker-based treatments are also available, such as for patients with germline BRCA mutation and programmed death-ligand 1 (PD-L1) expression in the tumor (Anders and Carey, 2022). For patients with PD-L1-positive metastatic TNBC, the anti-PD-1 antibody pembrolizumab combined with chemotherapy is a standard first-line treatment based on the results from the KEYNOTE-355 trial (Cortes et al., 2020). For patients with germline BRCA mutations, the poly (ADP-ribose) polymerase (PARP) inhibitors olaparib or talazoparib are recommended (Barchiesi et al., 2021).

Despite the availability of these targeted agents for metastatic TNBC, chemotherapy remains the standard-of-care treatment for PD-L1-negative TNBC without BRCA mutation (Anders and Carey, 2022). Given the relatively low prevalence of PD-L1-positivity and BRCA mutation in TNBC (about 40% and 10–20% respectively), multiple new therapeutic targets are under investigation in preclinical and clinical studies (Pogoda et al., 2020; Torres and Emens, 2022). Currently, the most promising treatment for metastatic TNBC is immunotherapy involving blockade of immune checkpoints, such as PD-(L)1 and lymphocyte-associated gene 3 (LAG3), in combination with other therapeutic agents (Huppert et al., 2022). Immune checkpoint molecules are membrane proteins that act as regulators of the immune system, which suppress antitumor immunity in cancer (He and Xu, 2020). In particular, CD47 is an immune checkpoint molecule that can inhibit phagocytosis of cancer cells by tumor-associated macrophages (TAMs), and CD47 blockade has been found to synergize with chemotherapy cabazitaxel in preclinical models of TNBC (Cao et al., 2021).

The second most prevalent immune cell subtype in TNBC, TAMs have a diverse functional spectrum (Karaayvaz et al., 2018; Williams et al., 2016). Based on the expression of functional markers, TAMs can be categorized into two general subtypes: classically activated (M1) and alternatively activated (M2) macrophages. Whereas M1-like macrophages exhibit pro-inflammatory functions such as phagocytosis of cancer cells, M2-like macrophages play important roles in facilitating tumor metastasis and other immuno-suppressive activities. Through cytokine secretion, TAMs can reduce effector T cell functions, facilitate regulatory T cell expansion, and induce immune checkpoint expressions, thus promoting an immuno-suppressive tumor microenvironment (Santoni et al., 2018). Clinically, TNBC is highly infiltrated by TAMs, which are associated with higher risk of distal metastasis and poor prognosis (Yuan et al., 2014). Moreover, M1-and M2-like macrophages have been shown to correlate with tumor response to immunotherapy and neoadjuvant chemotherapy in TNBC (Arole et al., 2021; Zhang et al., 2021b). Overall, TAMs serve as major contributors to tumor progression and are correlated with reduced survival in multiple cancer types (Larionova et al., 2019; Quail and Joyce, 2013). Therefore, promising treatments that target macrophages are being designed to reduce their recruitment, reprogram them toward the M1 phenotype, and block immune checkpoints that inhibit phagocytosis by TAMs (Quail and Joyce, 2017; Xie et al., 2022). Nonetheless, translation of promising preclinical studies into clinical benefits is not always successful (Duan and Luo, 2021).

In parallel with the clinical efforts, quantitative systems pharmacology (QSP) models can be developed in immuno-oncology to mechanistically and quantitatively investigate the course of disease progression in response to various treatments of interest (Bradshaw et al., 2019). By integrating the mechanistic knowledge from various disciplines, such as drug pharmacokinetics and pharmacodynamics (PK/PD), systems biology, and physiology, the models can explore the behavior of the system as a whole and uncover hidden emergent properties that may potentially point to new therapeutic applications (Pichardo-Almarza and Diaz-Zuccarini, 2016). Because of the large scope and complexity of QSP models, challenges remain during workflow standardization of model development and assessment, including model reproducibility, calibration, and validation (Bai et al., 2021).

In the past few years, we have developed and expanded a large-scale QSP platform for the analyses of immune checkpoint inhibitors and bispecific T cell engagers in combination with other agents in non-small cell lung cancer (Jafarnejad et al., 2019; Sové et al., 2020), colorectal cancer (Ma et al., 2020a; 2020b) and breast cancer (Wang et al., 2020a, 2021). We have also combined the QSP model with a spatial agent-based model of tumor to describe spatial heterogeneity of the tumor microenvironment (Gong et al., 2021; Zhang et al., 2021a). Here, by integrating a macrophage module into our previously published QSP platform (Wang et al., 2020a, 2021), we are able to investigate the impact of TAMs on the cancer-immune cell interactions and provide a computational framework to predict clinical response to macrophage-targeted agents based on preclinical data. We aim to demonstrate our calibration and validation steps to integrate the new macrophage module and show that the expanded model retains its predictive power with recalibrated virtual patient population using recently published data. Besides those inherited from our previous study (see STAR methods), new data includes intratumoral cytokine concentration measured directly from TNBC tumor biopsy samples (Autenshlyus et al., 2021), M1/M2 macrophage ratio from omics data analysis (Reynolds et al., 2017), and density of TAMs estimated by digital pathology analysis (Yang et al., 2018).

## Results

### Integrating the macrophage module into the QSP platform

**Figure 1:**
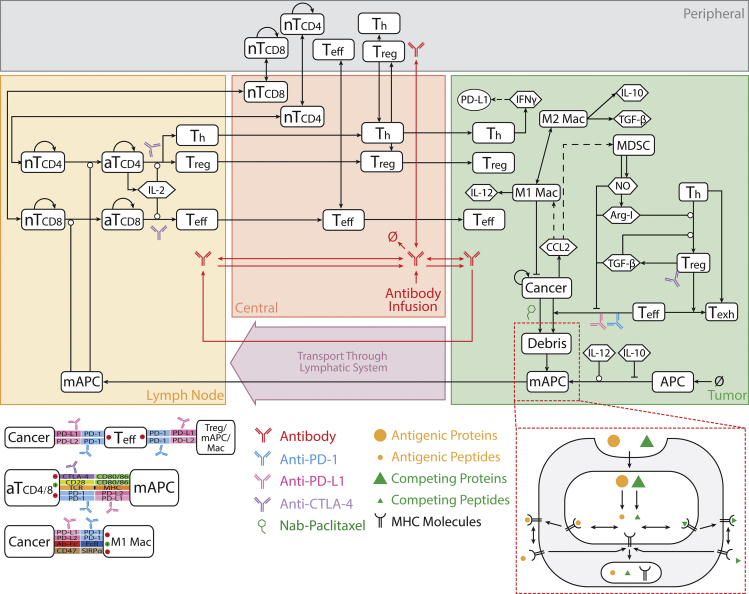
QSP model diagram The model is comprised of four compartments: central, peripheral, tumor, and tumor-draining lymph node, which together describe cycles of immune activation in lymph nodes, T cell trafficking to the tumor, killing of cancer cells, immune evasion, and antigen release and lymphatic transport (Wang et al., 2021). nT, naive T cell; aT, activated T cell; NO, nitric oxide; Arg-I, arginase I; Treg, regulatory T cell; Teff, effector T cell; Th, helper T cell; Mac, macrophage; mAPC, mature antigen-presenting cell. Cytokine degradation and cellular clearance are omitted in the figure.

In our previously published QSP platform for TNBC immunotherapy, modules were built to investigate the dynamics of T cells (i.e., effector T cell, regulatory T cell, helper T cell), antigen-presenting cells (APCs), cancer cells, tumor-specific neoantigens and tumor-associated self-antigens, immune checkpoints, myeloid-derived suppressor cells (MDSCs), and therapeutic agents, respectively. Figure 1 shows the interactions between TAMs and the pre-existing model species. As described in our previous study (Wang et al., 2021), the model includes four main compartments: central compartment describes circulation of therapeutic agents and immune cells between the bloodstream and the other parts of the body; peripheral compartment represents peripheral organs/tissues with naive T cell maintenance (den Braber et al., 2012); lymph node compartment represents tumor-draining lymph nodes at immediate downstream of a breast tumor, where T cell activation occurs; tumor compartment describes dynamics of cancer cells, activated T cells, APCs, and MDSCs. Sub-compartments were built to describe immune synapses between T cells and cancer cells/APCs, as well as the endosomal/surface space within/on APCs for antigen possessing and presentation.

**Figure 2:**
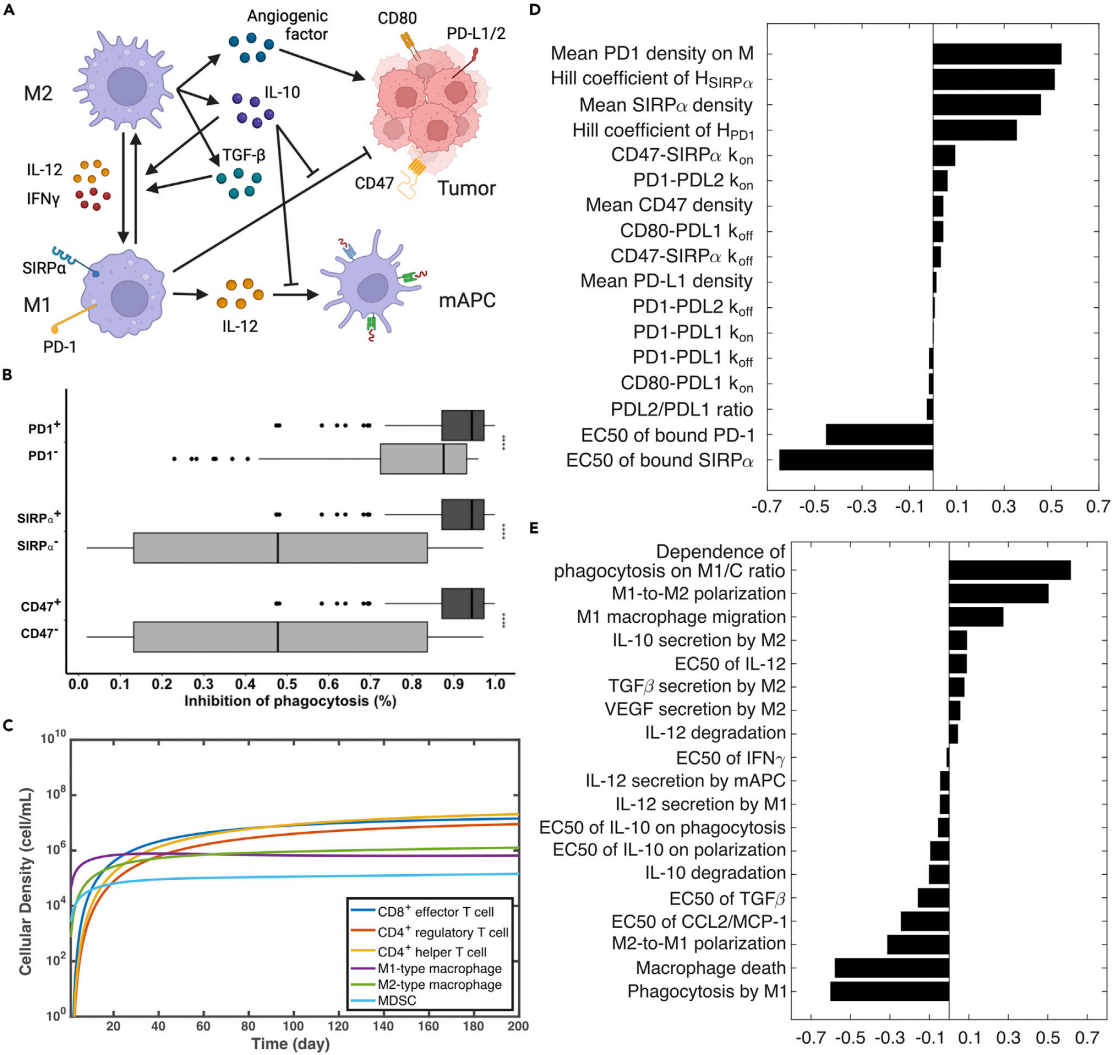
Integration of the macrophage module into the QSP model (A) Macrophage module diagram. Created with BioRender.com. (B) Model-predicted overall inhibitory effect on phagocytosis by immune checkpoint molecules $\left(H_{Mac,C}\right)$. Experimentally measured increases of phagocytosis activity are compared with the decrease of phagocytosis rate because of checkpoint interactions in the model $\left(1-H_{Mac,C}\right)$. Statistical significance was calculated by Wilcoxon test. (C) Time-dependent profile of T cell subsets, macrophages, and myeloid-derived suppressor cells (MDSC) from baseline simulation. (D) Global sensitivity analysis on phagocytosis submodule. Partial-rank correlation coefficients (PRCC) are reported to test associations between the overall inhibitory effect of checkpoint molecules on phagocytosis and parameters of interest. (E) Global sensitivity analysis on macrophage module. PRCC values are reported to test associations between the tumor volume and parameters of interest.

The modifications implemented in this study are two-fold. Mechanistically, dynamics of TAMs are added in the macrophage module with a phagocytosis submodule describing checkpoint interactions in the immune synapse between cancer cells and TAMs. We also updated binding between CD80 and PD-L1 in the checkpoint module, which were recently found to only occur on cellular surface of the same cell (in *cis*) (Zhao et al., 2019b). In terms of parameterization, majority of the parameter values are inherited from our previous estimation with a few updates based on recent data (Tables S1 and S2). Parameter estimation for the macrophage module is described below. Macrophage polarization is an estimate of macrophage activation at a given time point, which is a plastic process and plays diverse roles in nonresolving inflammation like cancer (Murray, 2017). Here, we adopted the classical dichotomy model for macrophages, which categorizes them into two subtypes: M1-and M2-like macrophages (Wei et al., 2021). At the beginning of tumor development, monocytes are recruited first by CCL2 into the tumor where they differentiate to pro-inflammatory M1-like macrophages (Yang et al., 2014). We assume that the differentiation occurs more rapidly than recruitment, so the entire process is modeled by a CCL2-mediated recruitment of M1-like macrophages (Equation S50). Once recruited, TAMs can be polarized toward pro- (M1) or anti-inflammatory (M2) macrophages by T cell- and tumor-derived cytokines. Figure 2A shows the cytokines that participate in the polarization process. IL-12 and IFNγ shift TAMs toward M1-like phenotypes while IL-10 and TGF-β shift TAMs toward M2-like phenotypes.

The model calibration started by estimating the recruitment and polarization rates of TAMs. The recruitment rate was estimated by the median density of TAMs measured in tumor nests from patients with basal-like breast cancer, a subtype similar to TNBC, by immunohistochemistry using a pan-macrophage marker, CD68 (Yang et al., 2018). The reported 2D density was converted to 3D density using a stereological equation (Mi et al., 2020). Assuming that M1-to-M2 polarization can occur as fast as two days, we estimated the rate of M2-to-M1 polarization to match the M1/M2 macrophage ratio with the median of clinically measured values in basal-like breast cancer. The clinical measurements were extracted from the ISB Cancer Genomics Cloud (ISB-CGC) (Reynolds et al., 2017), where proportions of M1-and M2-like macrophages among immune cells in breast tumors were determined using CIBERSORT (Newman et al., 2015). Notably, the calibration aimed to fit the model-predicted values (i.e., cellular density, M1/M2 ratio, etc.) at the time point when the tumor reached the median pre-treatment lesion size (Table S1), given that majority of the clinical measurements were taken upon diagnosis.

The functions of M1-like macrophages are facilitating APC maturation via IL-12 secretion, and phagocytosis of cancer cells. As IL-12 is the major cytokine for mediating APC maturation and subsequent T cell activation, it is mainly secreted by M1-like macrophages and mature APCs (mAPCs). The secretion rate of IL-12 by macrophages is estimated by the IL-12 level produced by TAMs isolated from solid tissue of human ovarian carcinoma and macrophages during the wound healing process in humans (Clough et al., 2007; Sica et al., 2000), and the secretion rate by mAPCs is roughly ten times higher upon antigen stimulation (Heufler et al., 1996). Rates of cytokine secretion by M2-like macrophages (i.e., IL-10, TGF-β, and VEGF) are estimated similarly (see STAR methods). Further, macrophage-mediated phagocytosis is a slow process that not only takes days to complete but is also inhibited by IL-10 and CD47 and PD-L1, two immune checkpoints on cancer cells (Bian et al., 2016; Gordon et al., 2017; Pan et al., 2020). To model the overall inhibitory effect of checkpoint molecules on phagocytosis, we built a submodule that incorporates interactions that impinge on these checkpoints, including PD-L1 upregulation by IFNγ, *trans* interactions between CD47 and SIRPα, PD-1 and PD-L1, and *cis* interaction between PD-L1 and CD80 on the surface of cancer cell. We also incorporated checkpoint blockade by specific antibody drugs (e.g., anti-CD47 and anti-PD-L1). The overall inhibitory effect of PD-1 and SIRPα on phagocytosis is calculated by Hill functions via Equations S42,S53 and S54.

To parameterize the submodule, density of checkpoint molecules, binding affinity between ligands and receptors, and Hill function parameters were required. 2D densities of CD47 and its receptor on macrophages, SIRPα, were estimated by *in vitro* assays (Morrissey et al., 2020; Subramanian et al., 2006). Because the absolute number of PD-L1 on cancer cells was not available from literature, PD-L1 density on cancer/immune cells in the tumor was estimated based on the *in vitro* measurement on mature dendritic cells and the percentage of tumor/immune cells (45%) that had concurrent PD-1 and PD-L1 expression in TNBC (Cheng et al., 2013; Gatalica et al., 2014; Wang et al., 2021). It was known that CD80 is expressed in TNBC cell lines (Navarrete-Bernal et al., 2020), which could interact with PD-L1 in *cis*, so the density of CD80 on cancer cells was also estimated by that on mature dendritic cells. Further, binding affinities between all ligands and receptors were available from the literature (Cheng et al., 2013; Jansson et al., 2005). Assuming a Hill coefficient of two for all checkpoint-mediated inhibitions, other parameters, such as the PD-1 density on TAMs and half-saturation constants were estimated to match the experimentally measured phagocytosis activity when one of the checkpoints was absent or blocked by an antibody. Figure 2B shows the overall inhibitory effect of checkpoints based on the checkpoint expression status from model simulation after parameterization. Simulated data points were generated by randomly sampling the density of checkpoint molecules (according to the distributions in Table S1) and calculating the overall inhibitory effect with calibrated Hill function parameters using Equation S54. Negative checkpoint status was simulated by setting the corresponding checkpoint density to 0. Consistent with published experimental studies, the model predicted that phagocytosis of cancer cells by TAMs was about eight times higher when treated by an anti-CD47 antibody (Willingham et al., 2012) and 2–3 times higher when compared with PD-1-negative TAMs (Gordon et al., 2017).

Figure 2C shows the dynamics of immune cell subsets in the tumor upon model calibration. Pro-inflammatory macrophages and MDSC are first recruited into the tumor by CCL2, which is followed by the infiltration of CD8^+^ effector and CD4^+^ helper T cells and eventually accumulation of regulatory T cells. Because of a higher M2 polarization rate, more M1-like macrophages are polarized to M2-like macrophages than M2 to M1, which results in a lower M1/M2 ratio as the tumor grows. By comparison, density of M1-like macrophages and CD8^+^T cells reach 1e5 cell/mL at day 2 and 14, respectively. Sensitivity analysis shows that the most influential parameters are densities of PD-1 and SIRPα on TAMs, Hill function parameters for inhibition on phagocytosis (Figure 2D); and rates of TAMs recruitment, polarization, and phagocytosis for tumor growth (Figure 2E).

### Revisiting analysis of the IMpassion130 trial with the macrophage module

After integrating TAMs into the QSP platform, we investigated if the model retains the predictive power on efficacy prediction for clinical trials. To this end, we performed in silico virtual clinical trials using the same approach as our previous analysis of atezolizumab and nab-paclitaxel treatments (Wang et al., 2021), but with a recalibrated virtual patient population. We inherited the virtual patient distributions from our previous analysis (Wang et al., 2021), which, along with the newly added modules and parameters, were (re)calibrated based on recently published data, such as intratumoral cytokine concentration, density of TAMs, and checkpoint expression. Due to the nonlinear nature of the QSP model, the outputs (e.g., CD8/Treg and CD4/Treg ratios) from simulation with median parameter values did not correspond to the median characteristics of the virtual patient population. Therefore, the descriptive statistics (i.e., median, standard deviation, upper and lower bounds) for parameter distributions were manually adjusted within the physiologically reasonable ranges so that the median characteristics of the virtual patient population matched the clinical measurements. Table 1 lists the model-predicted pre-treatment characteristics of the virtual patient population in comparison with clinically measured values from different sources. 3D cellular densities were either directly reported by clinical studies (e.g., MDSC) or estimated from 2D densities as described above (e.g., TAMs, T cells). Intratumoral cytokine concentrations were measured in a cluster analysis of TNBC biopsy samples (Autenshlyus et al., 2021).

**Table 1:** Comparison between virtual population statistics and clinical measurement of cytokine concentration and cellular density

Cellular Density (cell/mL)	VP Median	VP Range	Measured Median	Measured Range	Reference
CD8^+^ T cell	1.7e7	1.5e5–3.1e8	1.2e8	7.6e6–3.4e8	(Cimino-Mathews et al., 2016)
CD4^+^ T cell	2.3e7	4.0e5–5.0e8	1.4e8	6.1e6–1.2e9	
FoxP3^+^ Treg	7.7e6	1.6e5–1.4e8	5.5e7	5.8e6–1.7e8	
CD8/FoxP3	1.95	0.03–18.8	2.1	1.0–5.2	
CD4/FoxP3	2.64	1.04–19.4	2.6	0.9–11.0	
MDSC	1.7e5	1.1e4–1.7e6	1.6e5	4.3e4–5.3e6	(Diaz-Montero et al., 2009)
Macrophages	2.2e6	2.0e5–2.4e7	2.1e6	3.2e5–1.3e7	(Yang et al., 2018)
M1/M2	0.45	0.21–3.60	0.40	0–2.54	(Reynolds et al., 2017)

**Cytokine Concentration (pg/mL)**	**VP Median**	**VP Range**	**Measured Mean**	**SE**	**Reference**

IL10	19.7	0.96–270	I (n = 23): 6.47; II (n = 9): 16.60; III (n = 16): 29.93	I: 1.09; II: 3.63; III: 6.98	(Autenshlyus et al., 2021)
IFNγ	13.1	0.06–449	I: 16.56; II: 32.96; III: 9.38	I: 3.00; II: 8.21; III: 1.44	
CCL2	2203	333–14928	I: 2320.78; II: 4454.86; III: 11512.85	I: 344.58; II: 2249.72; III: 1457.94	

Cytokines were measured from cultured biopsy samples in groups I-III divided by multivariate cluster analysis in ref (Autenshlyus et al., 2021).

Treatment-related parameters were then recalibrated based on results from a phase 1 clinical trial of atezolizumab monotherapy (Emens et al., 2019) and the placebo comparator arm (placebo + nab-paclitaxel) of the IMpassion130 trial (Schmid et al., 2018). We generated about 1000 virtual patients and conducted in silico virtual clinical trials of atezolizumab and nab-paclitaxel monotherapies using the same dose regimens from the corresponding clinical studies as the dose schedules had been optimized to balance efficacy and toxicity. Specifically, a 1200 mg dose of atezolizumab was administered every 3 weeks (Q3W), or a 100 mg per m^2^ body surface area dose of nab-paclitaxel was administered on day 1, 8, 15 every 28 days (Q3/4W). During the clinical trial simulation, tumor size was recorded every 8 weeks by the model, which corresponds to the frequency of tumor size measurement in the clinical studies. According to the model-predicted tumor size/diameter, response status was determined using RECIST 1.1, assuming that a minimum duration of 8 weeks is required for the categorization of stable disease (Eisenhauer et al., 2009). Clinically reported objective response rates (ORR) and duration of response (DOR) were used to calibrate treatment-related parameters. Table S1 summarizes the calibrated parameter distributions for virtual patient generation.

**Figure 3:**
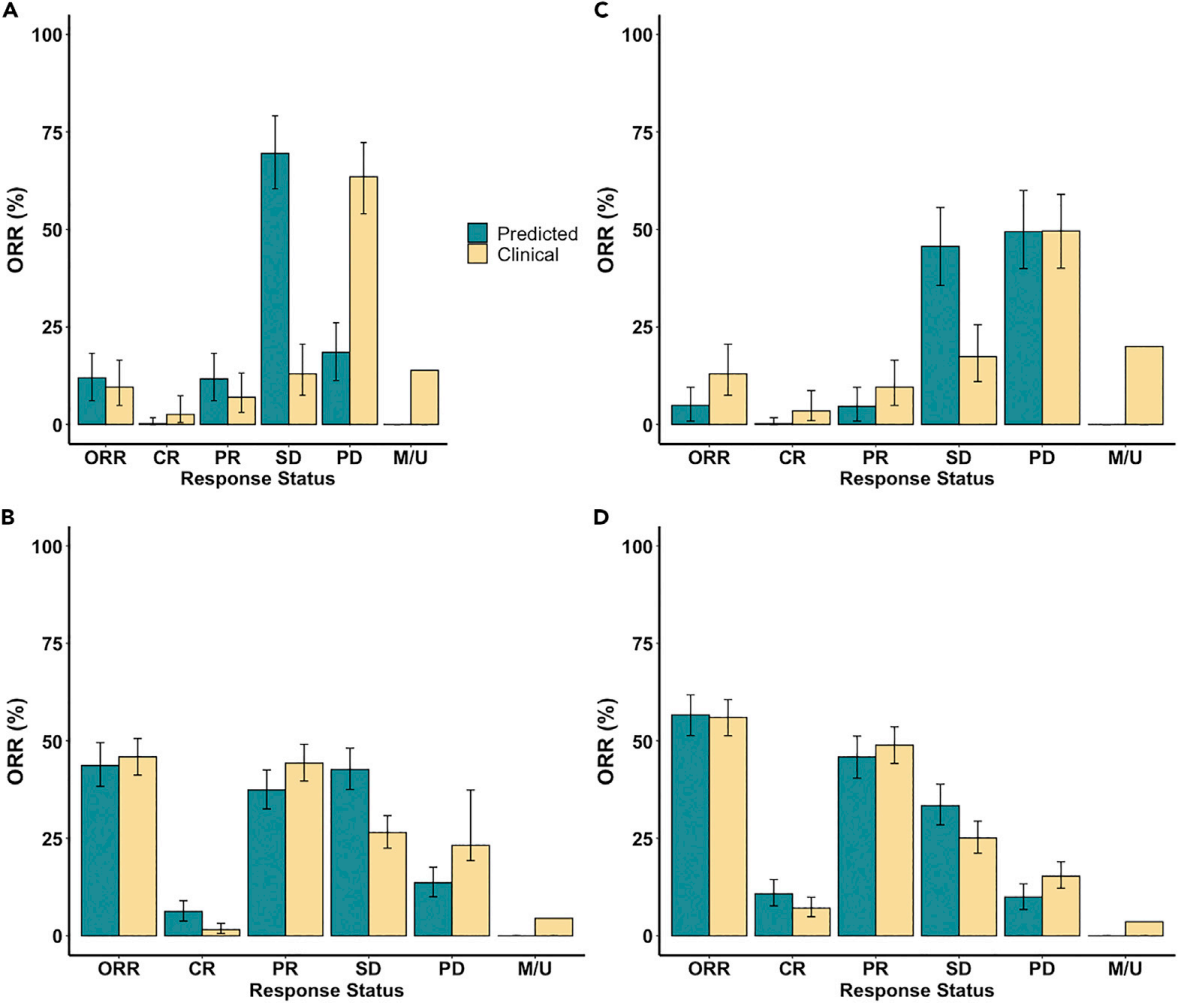
Response status comparison between model prediction and clinical results in (A) atezolizumab monotherapy by RECIST 1.1, (B) nab-paclitaxel monotherapy by RECIST 1.1, (C) atezolizumab monotherapy by irRC, and (D) combination treatment by RECIST 1.1 Model predictions are presented with 95% bootstrap confidence intervals, while clinical results are reported with 95% Clopper–Pearson confidence intervals (Emens et al., 2019; Schmid et al., 2018). Virtual patient who has a tumor smaller than 2 mm is assumed to be a complete responder by the model. ORR, objective response rate. CR, complete response. PR, partial response. SD, stable disease. PD, progressive disease. M/U, missing or unevaluable disease.

**Figure 4:**
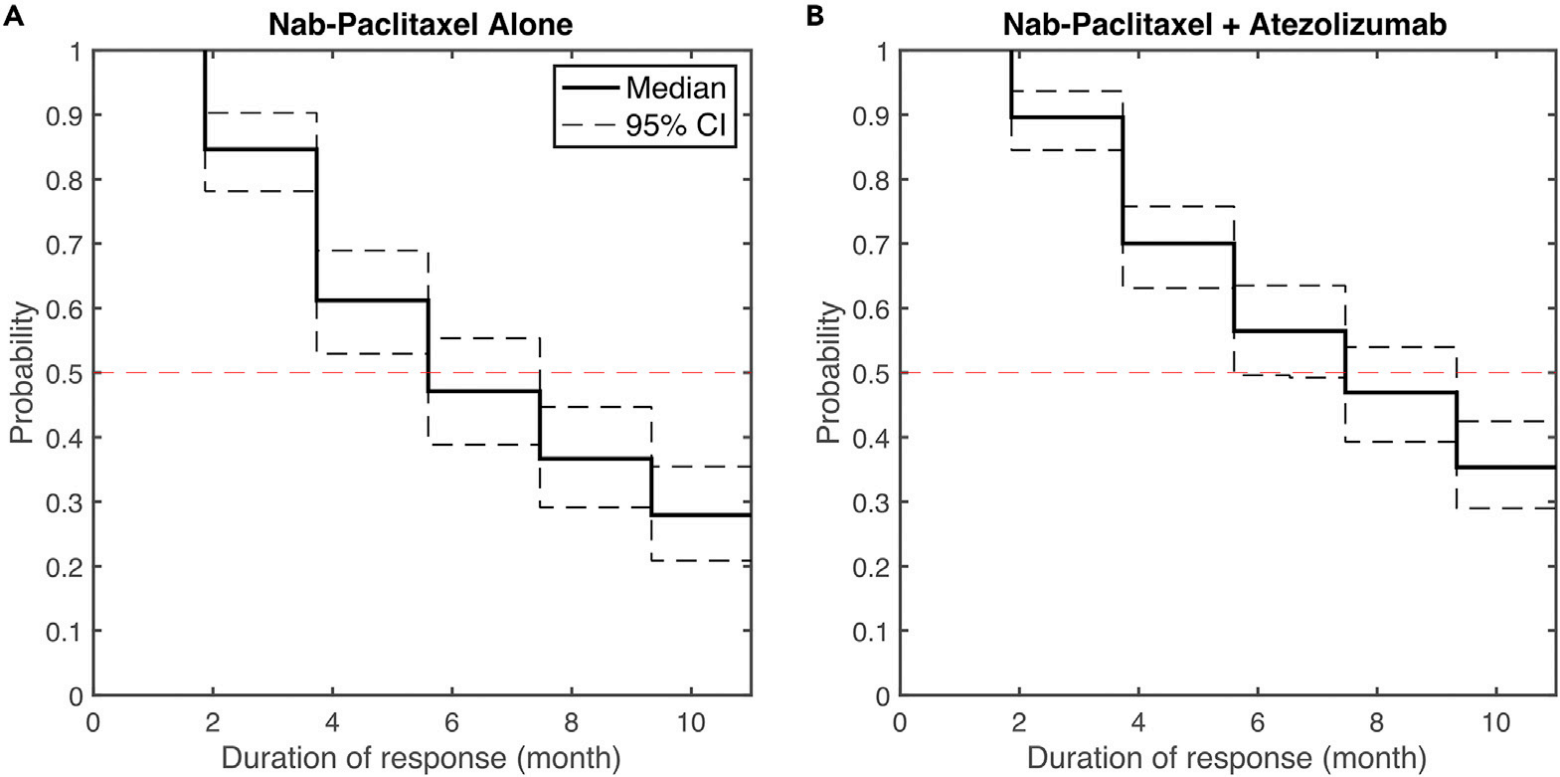
Kaplan-Meier curve of model-predicted duration of response in (A) nab-paclitaxel monotherapy and (B) combination treatment of nab-paclitaxel and atezolizumab Duration of response is defined as the time from the achievement of a response to progression. The median durations of the response with 95% bootstrap confidence intervals are 5.6 (5.6–7.5) and 7.5 (5.6–9.3) months, respectively.

Figures 3A and 3B compares the model-predicted efficacy of atezolizumab or nab-paclitaxel alone using calibrated virtual patient population with the results from clinical trials. Here, we report the median and 95% confidence intervals using bootstrap sampling of the virtual patient population. The bootstrap sampling size was set to the number of patients enrolled in the corresponding clinical study. Similarly, Figure 4A shows the Kaplan-Meier curve for model-predicted duration of response on nab-paclitaxel monotherapy. Overall, the model-predicted ORR and DOR are consistent with the clinical results, which fall within the 95% confidence intervals of model predictions. When further characterizing virtual patients into subcategories (i.e., complete/partial response, progressive/stable disease), we observed a shift of more progressive disease to more stable disease in model prediction compared to clinical results. We hypothesized that this shift was because of our assumption on the minimum duration for stable disease (i.e., 8 weeks) and the lack of model prediction of new metastatic lesion, which would be categorized as progressive disease by RECIST criteria (Eisenhauer et al., 2009). As a result, we recategorized the response status in the atezolizumab monotherapy using immune-related response criteria (irRC), by which patients are considered to have partial response or stable disease even if new lesions are present, as long as the respective thresholds of response are met (Wolchok et al., 2009). Figure 3C shows that irRC alleviated the overestimation of stable disease by the model. Interestingly, the model-predicted ORR is lower than the clinically reported value when using irRC, because of its stricter criteria for partial response (i.e., a 50% tumor shrinkage is required), which suggests that more patient data are needed for model calibration to improve its predictive power.

**Figure 5:**
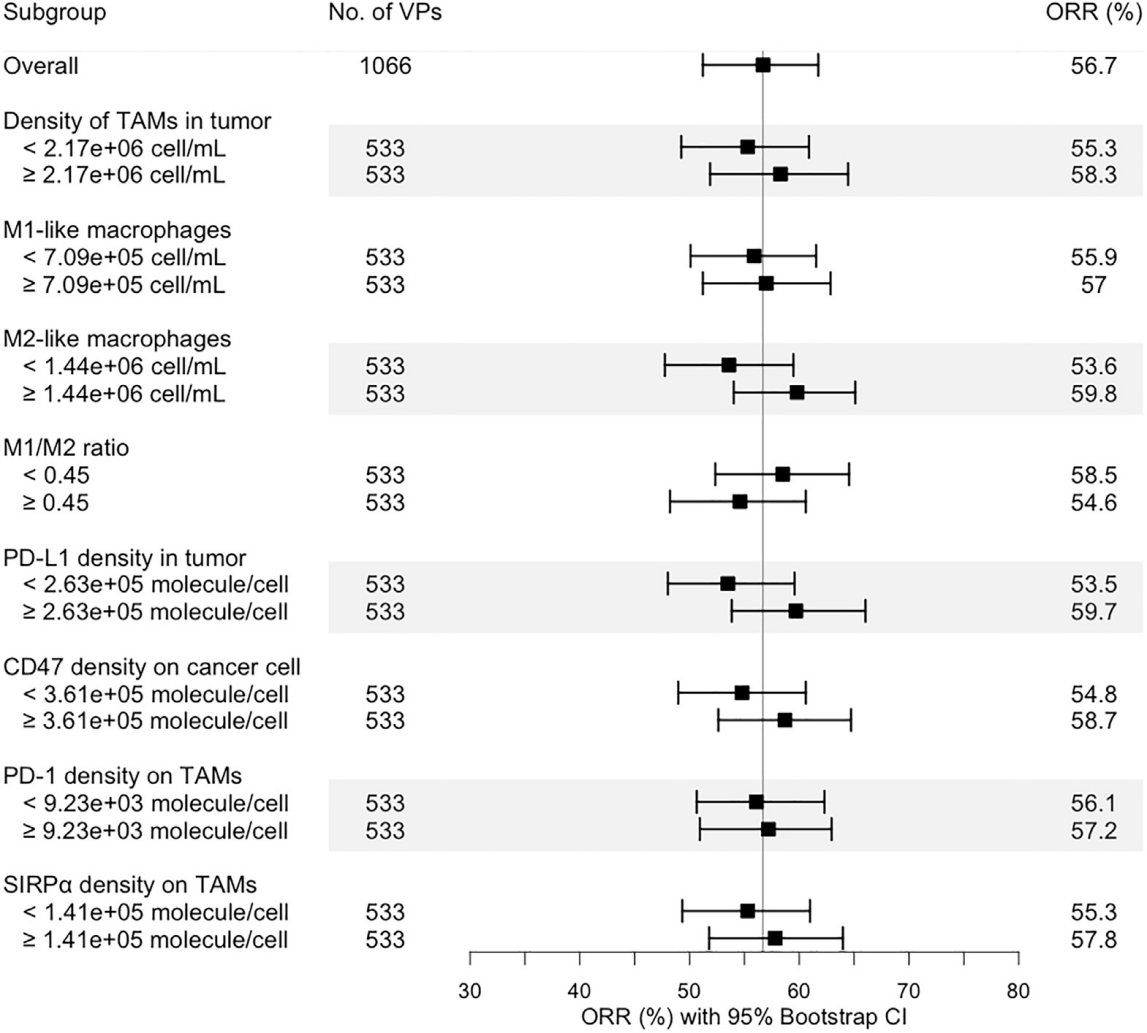
Subgroup analysis of the in silico clinical trial of atezolizumab and nab-paclitaxel The virtual patient population was divided into eight subgroups based on the pre-treatment values of selected characteristics, and the objective response rates in each subgroup were calculated with 95% bootstrap confidence intervals.

To validate our model structure, we conducted an in silico trial of combination treatment using atezolizumab and nab-paclitaxel with the recalibrated virtual patient population (Table S1). According to the dose regimen in the experimental arm of the IMpassion130 trial, 800 mg atezolizumab was administered every 2 weeks (Q2W) with 100 mg/m^2^ nab-paclitaxel administered Q3/4W. Figures 3D and 4Bshow that the model-predicted ORR and DOR for combination treatment of atezolizumab and nab-paclitaxel are consistent with clinically reported values. In our previous analysis, a zero complete response rate was predicted in atezolizumab monotherapy with a limited increase when atezolizumab was combined with nab-paclitaxel (Wang et al., 2021). With newly incorporated dynamics of TAMs, the increase in complete response in the combination treatment is more consistent with clinical observation, likely because of the restoration of phagocytosis activity by TAMs upon PD-L1 blockade. To further investigate the role of TAMs in this combination therapy, we performed a subgroup analysis by dividing the virtual patients into subgroups by the medians of model variables of interest and calculating the ORR in each subgroup. Figure 5 suggests that density of TAMs and their subsets, as well as checkpoint expressions on TAMs and cancer cells, may not be predictive biomarkers for this combination regimen, as the confidence intervals overlap between subgroups. As atezolizumab and nab-paclitaxel show additive effect when they are combined in clinical trials and our simulations, majority of the responders in the combination regimen are most likely because of the addition of chemotherapy. As a result, the level of tumor-infiltrating lymphocytes and PD-L1 may not be as predictive as in anti-PD-L1 monotherapy, which relies on restoration of anti-tumoral activity by immune cells through blockade of the immune checkpoint.

**Figure 6:**
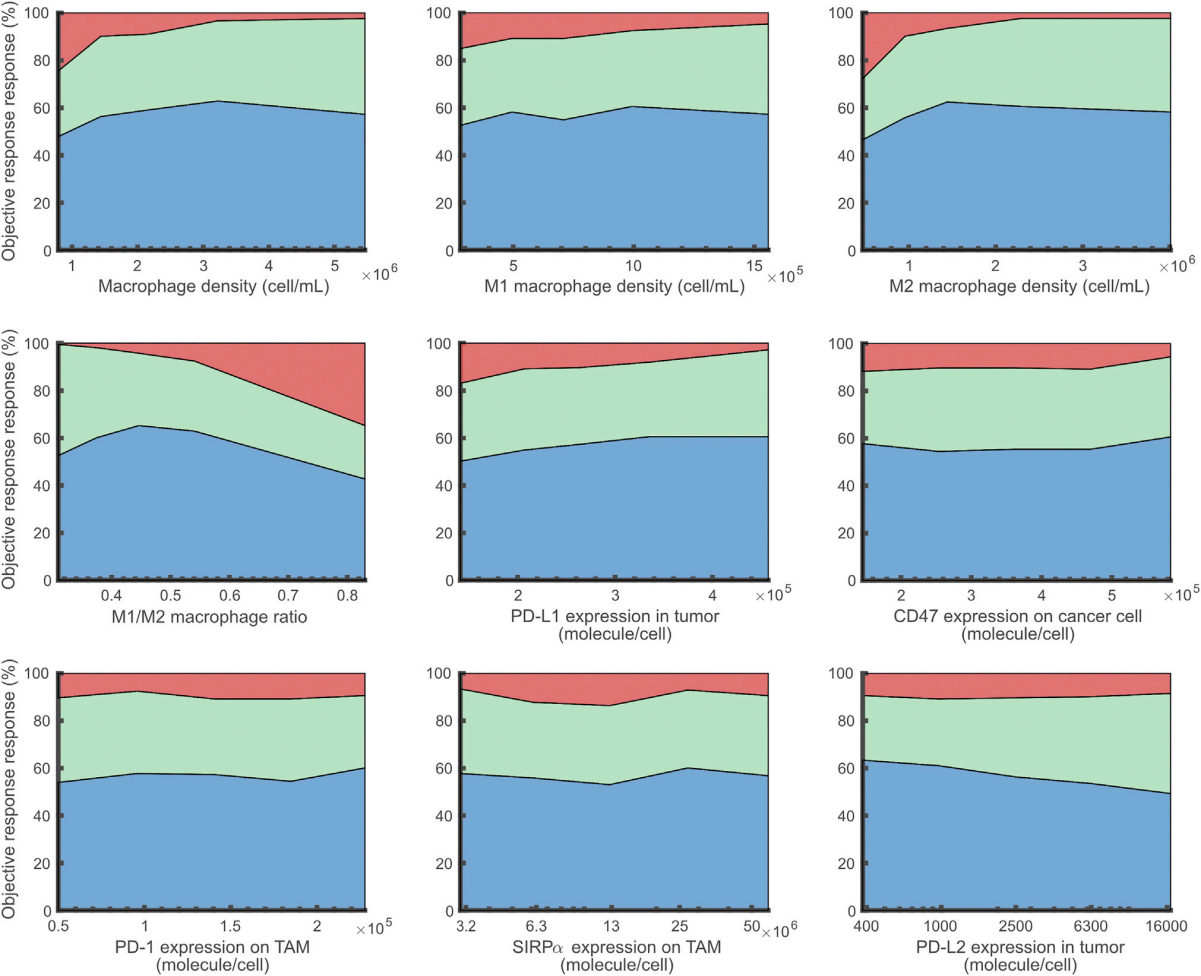
Effects of model variables on response status For each variable, the virtual patient population was sorted by the pre-treatment variable level in ascending order, and evenly divided into five subgroups. The response status of each subgroup in the combination therapy is plotted against the corresponding median variable level. Blue represents partial or complete response. Green represents stable disease. Red represents progressive disease. Related to Figure S1.

We further divided the virtual patients into five subgroups based on the level of each variable of interest and calculated the ORRs in each subgroup. In Figure 6, the differences in ORR were greater than 10% when the subgroups were generated based on density of M2-like macrophages, M1/M2 ratio and expressions of PD-L1, SIRPα, and PD-L2. Specifically, ORR increases as PD-L1 level increases which meets our expectation because a high PD-L1 expression is correlated with high T cell infiltration and thus better response to the anti-PD-L1 treatment (Kitano et al., 2017). On the other hand, ORR decreases as SIRPα and PD-L2 densities decrease, suggesting their potential roles in treatment resistance. Surprisingly, ORR decreases as density of M2-like macrophages and M1/M2 ratio increase, even though M2 macrophages exhibit only immunosuppressive activities and have shown a positive correlation with tumor growth without treatment (Figure 2E). This prediction could potentially result from the correlation between M1-like macrophages and immunosuppressive species, including TGF-β that facilitates polarization to M2 phenotype (Figure S1). This correlation was also observed in the clinical analysis by (Oshi et al., 2020). Also, the genetic markers used for subtype classification of TAMs differ across studies, which could impact the correlations observed in clinical analyses (Newman et al., 2015; Zhang et al., 2021b). Therefore, there is a need for better understanding of TAMs in the context of treatments (Xiang et al., 2021). Importantly, we have demonstrated here that when virtual patients are calibrated based on population-level data from preclinical studies and clinical trials of monotherapy, the QSP model can make reliable efficacy prediction for the combination treatment.

## Discussion

We have here expanded our previously published QSP platform by integrating a macrophage module to investigate the dynamics of TAMs in TNBC. Model calibration, which involves both the new and the pre-existing modules as well as the virtual patient distributions, were described in detail above and in STAR methods. In practice, the QSP platform was originally built with seven modules, each of which was written by a MATLAB script with built-in functions of SimBiology Toolbox (Sové et al., 2020). New modules were then developed as independent user-defined MATLAB functions that integrate new model elements into the QSP model. In this way, modules can be readily added and removed according to the aims of the study. In fact, the QSP platform has been applied to predict tumor response to various treatments in multiple cancer types with different modules incorporated based on study objectives and data availability for model calibration. This high-usability feature greatly facilitates the wide application of such a platform model in immuno-oncology translational research and drug development (Chelliah et al., 2021).

As discussed in this study, the macrophage module allowed us to investigate the roles of M1-and M2-like TAMs during immunotherapy and chemotherapy in TNBC and improved the ORR predictions. In particular, PD-1 is expressed on both TAMs and effector T cells (Teff) so that both cytotoxic activity of Teff and phagocytosis are inhibited by PD-1-PD-L1/2 interactions, providing an additional target for anti-PD-1/L1 treatments (Gordon et al., 2017). Further, we previously hypothesized that PD-L1 expressed on T cells can interact with CD80 on APC and thus block the co-stimulatory signals (i.e., CD28^−^CD80/86) during T cell activation. However, recent findings have suggested that only *cis* interactions occur between CD80 and PD-L1 (Zhao et al., 2019b). Also, PD-L1 heterodimerizes with CD80 and selectively weakens its interaction with CTLA-4 but not CD28, which provides a mechanistic explanation for the clinically observed synergistic effect of anti-PD-L1 and anti-CTLA-4 treatments (Zhao et al., 2019b). In addition to PD-1-mediated inhibition, CD47 also serves as an immune evasion mechanism developed by cancer cells. As shown above, phagocytosis by TAMs can be enhanced eight-fold in breast cancer when CD47 is blocked (Willingham et al., 2012), which suggests that it could be a potential target for cancer treatment, and the present QSP platform may serve as a tool to make prospective efficacy predictions for this emerging strategy and its combination with other checkpoint inhibitors.

In the model, we introduced M1-and M2-like TAMs as two extreme states. In reality, the states constitute a continuum, and we have recently developed systems biology models to describe the complexity of macrophage signaling under different conditions (Zhao and Popel, 2021; Zhao et al., 2019a, 2021). Using these modeling approaches, additional mechanistic detail could be accounted for in extensions of the macrophage module to further enrich the description of macrophage compositions in the tumor microenvironment. It should also be noted that the mechanistic inclusion of TAMs into the QSP platform is one of the several important aspects of the tumor microenvironment, as there are other cell components, such as natural killer (NK) cells, B cells, and fibroblasts that are known to influence cancer progression and treatment efficacy. However, such extensions of the platform have to be carried out step-by-step with careful iterative calibration and validation against cancer-specific experimental and clinical data (Makaryan and Finley, 2020; Makaryan et al., 2020). Although we have made every effort to make modules independent, integrating a module, as demonstrated in this study, is a meticulous process. New model reactions should be detailed enough to reflect the complexity of biological mechanisms that occur in reality, but simplified enough so that the model parameters can be identified by limited data. Once integrated, pre-existing parameters should be recalibrated to make sure that updated model outputs still fall within the physiologically reasonable ranges. This process can lead to a comprehensive mechanistic characterization of the tumor microenvironment that would provide rich therapeutic targets and valuable insights in the basic-to-translational quest in immuno-oncology research.

In silico virtual clinical trials, as we have explored here, is an emerging field of study in immuno-oncology (Chelliah et al., 2021). To capture the inter-individual heterogeneity of patients with metastatic TNBC, we estimated the virtual patient distribution according to pre/clinical data on TNBC or other breast cancer subtypes if TNBC data are not available. In addition, multi-omics and digital pathology data have proven useful when generating physiologically reasonable virtual patients, and in future such data can be further collected from TNBC and incorporated into our QSP platform (Lazarou et al., 2020; Zhang et al., 2021a). Notably, in the tutorial created by (Lazarou et al., 2020), they listed the multi-omics data that can be utilized to predict neoantigen binding affinity with MHC molecules, T cell receptor repertoire diversity, immune cell composition in lymph nodes, and more for QSP model calibration. More importantly, individual tumor growth trajectories from clinical trials could provide substantially more information for virtual patient generation than overall response rates. We also aim to match the simulation setting with the actual clinical trial setting, and we followed the same measurement frequency of tumor size and clinical criteria used in those trials when making efficacy predictions. Nonetheless, the current trial simulation workflow can be further improved by future efforts, as certain factors that lead to treatment discontinuation or categorization of progressive disease, such as appearance of new lesions, severe adverse events, and death, were not described during the in silico trials presented here. For example, unlike in chemotherapy where clinical response often ends by tumor progression led by chemo-resistance, the response in immunotherapy mostly ends when new lesions are found while tumor size is below the response threshold. As a result, the lack of model prediction of new lesions makes the model-predicted duration of response not comparable to the clinically reported value. Additionally, limited tumor detectability of imaging modalities used for tumor size measurement may also affect the response status. To be consistent with our previous analysis, we assume that patients with tumor smaller than 2 mm are categorized as complete responders. These factors should all be considered when interpreting the model predictions, especially for the subcategorization within responders and non-responders.

Although the accelerated approval of atezolizumab for PD-L1-positive metastatic TNBC has been withdrawn since our previous analysis, results from the IMpassion130 study provide useful information for model calibration and validation. The withdrawal of atezolizumab was because of two developments. First, clinical benefit was not observed in the confirmatory IMpassion131 trial (Miles et al., 2021), and the release of overall survival (Emens et al., 2021). Second, KEYNOTE-355 demonstrated both progression-free and overall survival benefit associated with the anti-PD-1 antibody pembrolizumab and chemotherapy, resulting in full regulatory approval for the first-line therapy of patients with metastatic PD-L1-positive TNBC. These results suggest that it may be useful to identify surrogate markers for survival prediction after treatments using the QSP platform, but patient and treatment-specific data are required (Gion et al., 2021). Also, visceral metastasis is correlated with poor survival outcomes in TNBC (Wang et al., 2020b). In the present model, we have only incorporated one tumor compartment that represents an average tumor lesion in metastatic TNBC, with two cancer clones (i.e., sensitive and resistant clones to nab-paclitaxel). Adding multiple tumor compartments that describe the formation and dynamics of metastatic lesions can better assist the study on tumoral heterogeneity and improve efficacy prediction by the model.

### Limitations of the study

One of the central factors that could influence the predictive power of in silico clinical trials using a QSP platform is the mechanistic hypotheses that aim to reflect the real biological processes. In our case, hypotheses are made based on our current understanding of the tumor-immune system, which is constantly updated by new experimental data, such as the *cis* interaction between CD80 and PD-L1 as discussed above. However, some biological processes are still not fully understood, such as the role of PD-L1 on T cells and mechanisms of action (MOA) of PD-1/L1 inhibitors (DeSousa Linhares et al., 2019; Kazanova and Rudd, 2021; Zhao et al., 2019b). This may result in a discrepancy between model prediction and clinical observation. In addition, some simplifications have to be made because of the lack of data during calibration of certain model components. For example, CD4^+^ helper T cells can be further categorized into multiple subsets (e.g., Th1, Th2, and Th17), each of which may play a unique role in the immune system. However, the lack of quantitative measurements of their cellular density in TNBC prevents them from being explicitly incorporated into the QSP model (Tay et al., 2021). Also, quantitative data were rarely reported for metastatic TNBC lesions, which are critical to study the tumoral heterogeneity (Pasha and Turner, 2021).

Another limitation is related to model parameterization, which has to account for the measurement uncertainty from experimental techniques, cross-study variability, and inter-individual heterogeneity. This is particularly reflected in virtual patient generation. In this study, we manually adjusted the descriptive statistics for each parameter distribution during virtual patient calibration, and randomly generated deviations from the median parameter values that are fitted to the experimental/clinical data. Given that the descriptive statistics and the distribution type can sometimes take more than one fixed set of values, this would have an impact on the model prediction as well as the parameter sensitivity. As a result, larger-than-reality ranges could be generated for parameters and model outputs, and without a sufficiently large dataset of relevant clinical measurements it is hard to apply a robust algorithm to narrow down the virtual patient population while preserving the desirable inter-individual heterogeneity. In addition, covariance between parameters was not considered because of the lack of patient data, as parameters are independently sampled during virtual patient generation, and thus could result in virtual patients that would not have existed in reality (Tivay et al., 2020). In summary, more precise selection criteria and screening methods need to be added into the overall virtual patient generation workflow, as additional data become available, to improve the predictive power of such model-based in silico trials in immuno-oncology research.

## STAR★Methods

### Key resources table

**Table:** 

REAGENT or RESOURCE	SOURCE	IDENTIFIER
**Software and Algorithms**		

MATLAB code for QSP model initiation and simulation	This paper	https://doi.org/10.5281/zenodo.6614447
MATLAB R2020b	MathWorks (Natick, MA)	https://www.mathworks.com/products/matlab.html
SimBiology	MathWorks (Natick, MA)	https://www.mathworks.com/products/simbiology.html
Symbolic Math Toolbox	MathWorks (Natick, MA)	https://www.mathworks.com/products/symbolic.html
RStudio v1.4.1717	RStudio, PBC (Boston, MA)	https://www.rstudio.com/products/rstudio/
Affinity Designer	Serif Europe (UK)	https://affinity.serif.com/en-us/designer/
WebPlotDigitizer	Ankit Rohatgi	https://automeris.io/WebPlotDigitizer

### Resource availability

#### Lead contact

Further information and requests for resources should be directed to and will be fulfilled by the lead contact, Hanwen Wang.

#### Materials availability

This study did not generate new materials or reagents.

### Method details

#### Model overview

The model comprises four compartments (central, peripheral, tumor, and tumor-draining lymph nodes) and ten modules, including a new macrophage module introduced in the present analysis. Each module describes the kinetics and dynamics of a major molecular or cellular species in the tumor microenvironment, such as cancer cells, T cells (i.e., effector T cells, regulatory T cells, helper T cells), and antigen-presenting cells (APCs). All modules from our previous analysis of the IMpassion130 trial (Wang et al., 2021) were inherited, modified, and recalibrated with the new macrophage module, using published data on triple-negative breast cancer (TNBC). The cellular dynamics involved in each module are described below, and the effects of molecular interactions (e.g., immune checkpoints and cytokines) are implemented using Hill functions $\left(e.g.,\;H_{A}=\frac{{\left[A\right]}^{n}}{{\left[A\right]}^{n}+A_{50}^{n}}\right)$ unless otherwise specified. The model comprises 152 ordinary differential equations (ODEs), 45 algebraic equations (i.e., repeated assignment rules), and 280 parameters. Model species, parameters, reactions, compartments, and algebraic equations are listed in Tables S2, S3, S4, S5 and S6. MATLAB scripts for model generation and in silico clinical trials are made available at https://doi.org/10.5281/zenodo.6614447 and https://github.com/popellab/QSPIO-TNBC.

#### Cancer module

To investigate the dynamics of tumor vasculature and the anti-angiogenic activity of chemotherapy, we adapted the modified Gompertzian model proposed by (Hahnfeldt et al., 1999). Because the tumor volume in our model is calculated by the total number of cancer cells in the tumor compartment, we modified the equations to describe cancer cell dynamics, where the tumor-carrying capacity, K, is translated to the maximal number of cancer cells supported by the tumor vasculature, *C*_*max*_.

(Equation S1) $$\frac{dC_{i}}{\mathrm{d}t}=k_{C,growth}C_{i}\;\log\left(\frac{C_{max}}{C_{\text{total}}}\right)-\left(k_{C,death}+k_{C,nabp}H_{nabp}\frac{\min\left(C_{total},K_{C,nabp}\right)}{C_{total}}+k_{M1,phago}\frac{{\left[Mac\right]}_{M1}}{{\left[Mac\right]}_{M1}+K_{Mac,C}C_{i}}\left(1-H_{Mac,C}\right)\left(1-H_{IL10,phago}\right)+k_{C,T}\frac{{\left[T_{eff}\right]}_{T}}{{\left[T_{eff}\right]}_{T}+K_{T,C}C_{i}}\frac{{\left[T_{eff}\right]}_{T}}{{\left[T_{eff}\right]}_{T}+K_{T,Treg}{\left[T_{reg}\right]}_{T}}\;\left(1-H_{TGF\beta ,Teff}\right)\left(1-H_{PD1,C}\right)\left(1-H_{MDSC}\right)\right)C_{i}$$

(Equation S2) $$\frac{dC_{max}}{\mathrm{d}t}=k_{K,g}C_{total}\frac{c_{vas}}{c_{vas}+c_{vas,50}}-k_{K,d}C_{max}{\left(C_{total}V_{cell}\right)}^{\frac{2}{3}}-k_{K,nabp}C_{max}\left[NabP\right]$$

(Equation S3) $$V_{T}\frac{dc_{vas}}{\mathrm{d}t}=k_{vas,Csec}C_{total}+k_{vas,Msec}{\left[Mac\right]}_{M2}-k_{vas,deg}c_{vas}V_{T}+k_{vas,nabp}C_{total}H_{nabp,vas}$$

Equation S1 describes the dynamics of cancer cells in each cancer clone, *C*_*i*_, added by the cancer module. The first term describes cancer cell proliferation with growth rate constant, *k*_*C*, *growth*_, total number of cancer cells in all cancer clones, *C*_*total*_, and the maximum number of cancer cells reflecting the tumor carrying capacity, *C*_*max*_. The second term describes the death of cancer cells because of apoptosis, cytotoxic action of nab-paclitaxel, phagocytosis, and effector T cells (Teff). Apoptosis, which is caused by natural cell death, is assumed to be a first-order reaction with the rate constant, *k*_*C*, *death*_ Cytotoxic activity of nab-paclitaxel is incorporated with the killing rate constant, *k*_*C*, *nabp*_, a Hill function with varying effective concentrations, *H*_*nabp*_, and the number of cancer cells that is accessible by nab-paclitaxel, *k*_*C*, *nabp*_. Rate of phagocytosis by TAMs depends on the phagocytosis rate constant, *k*_*M*1, *phago*_, ratio of effector and target cells, and the Hill functions for the inhibitory effects of checkpoint molecules, *H*_*Mac*, *C*_, and IL10, *H*_*IL*, *phago*_. Cancer cell killing by Teff is described by the killing rate constant, *k*_*C*, *T*_, the ratio of Teff and target cells, the ratio of Teff and regulatory T cells (Treg), and the Hill functions representing the inhibitory effects of PD-1, TGF-β, and molecules secreted by myeloid-derived suppressor cells (MDSC), *H*_*MDSC*_. The dependence of cancer cell killing by M1 macrophages and Teff on cellular ratios (i.e., M1/C, Teff/C, and Teff/Treg) is modelled by Hill-type dynamics with inhibition threshold parameters: *K*_*Mac*, *C*_, *K*_*T*, *C*_, and *K*_*T*, *Treg*_. (Robertson-Tessi et al., 2012). Equations for $H_{PD1,C}$, *K*_*Mac*, *C*_ and *H*_*MDSC*_ are shown in *Checkpoint module, Macrophage module,* and *MDSC module*, respectively.

Equation S2 calculates the maximal number of cancer cells supported by the tumor vasculature. The first term represents the growth of tumor vasculature induced by angiogenic factors, *c*_*vas*_, with a rate constant, *k*_*k, g*_. The second term represents the endogenous inhibition of existing tumor vasculature, such as endothelial cell death, with a rate constant, *k*_*k, d*_ (Hahnfeldt et al., 1999). The third term represents the inhibition of tumor vasculature because of nab-paclitaxel, with a dose-dependent rate, *k*_*K, nabp*_ (Mollard et al., 2017). The tumor angiogenic factor, *c*_*vas*_, is assumed to be secreted by cancer cell and M2-like macrophages and induced by nab-paclitaxel (Equation S3). The secretion rates are fitted to VEGF-A level measured in preclinical studies (Volk et al., 2008; Wu et al., 2010).

The tumor growth parameters (i.e., *k*_*C*,*growth*_, *k*_*K*, *g*_, *k*_*K*, *d*_, and *c*_*vas*50_) and the initial tumor carrying capacity, *C*_*max*_(0), are fitted to the tumor growth curve reported by preclinical studies using TNBC xenograft model. Fitting was performed with the simplex search method using a MATLAB function, *fminsearch*, to minimize the mean squared difference between observed and predicted values (Lagarias et al., 1998). Using the fitted values as medians, *k*_*C*, *growth*_ and *k*_*K*, *g*_ are varied assuming lognormal and uniform distributions, respectively, to capture the tumor heterogeneity. The median, 60th and 90th percentiles of the simulated tumor growth are plotted with the experimental measurements in Figure S2. *k*_*C*, *growth*_, *k*_*K*, *g*_, and *k*_*K*, *d*_ are then scaled up to human using Equation S4. The allometric scaling has been tested in models of various cancer types in human (Elassaiss-Schaap, 2010; Garcia-Cremades et al., 2019; Lindauer et al., 2017; West et al., 2002), while its robustness in translating growth of breast tumor xenografts to human needs to be investigated when clinical data become available.

(Equation S4) $$\theta_{human}=\theta_{mice}{\left(\frac{WT_{human}}{WT_{mice}}\right)}^{-0.25}$$

Tumor volume (tumor compartment capacity) is calculated by Equation S5 at each time step. *C*_*total*_, *T*_*total*_, and *M*_*total*_ represent the total number of cancer cells, T cells, and macrophages in the tumor; *C*_*x*_ and *T*_*exh*_ represent apoptotic cancer cell and exhausted T cell; *V*_*cell*_, *V*_*Tcell*_, and *V*_*Mcell*_ are the volumes of a single cancer cell, T cell, and macrophage, respectively. *V*_*e*_ is the volume fraction of the intracellular space in breast tumors, which was calculated assuming vascular and interstitial space occupy roughly 2 and 61% of the total tumor volume, respectively (Finley and Popel, 2012).

(Equation S5) $$V_{T}=\frac{1}{V_{e}}\left(V_{cell}\left(C_{total}+C_{x}\right)+V_{Tcell}\left(T_{total}+T_{exh}\right)+V_{Mcell}M_{total}\right)$$

#### T cell modules (Teff, Treg, and helper T cell)

##### Naïve T cell dynamics

Dynamics of naïve T cells are incorporated into the central (C), peripheral (P), and tumor-draining lymph node (LN) compartments. (Equation S6), (Equation S7), (Equation S8) describe dynamics of naïve CD4^+^ and CD8^+^T cells, where [*nT*]_*i*_ represents the average number of naïve CD4^+^/CD8^+^T cells of a single clonotype in the corresponding compartment.

(Equation S6) $$\frac{d}{\mathrm{d}t}{\left[nT\right]}_{C}=\frac{Q_{nT,thym}}{div}-Q_{nT,P,in}{\left[nT\right]}_{C}+Q_{nT,P,out}{\left[nT\right]}_{P}-Q_{nT,LN,in}{\left[nT\right]}_{C}+Q_{nT,LN,out}{\left[nT\right]}_{LN}-k_{nT,death}{\left[nT\right]}_{C}\;$$

(Equation S7) $$\frac{d}{\mathrm{d}t}{\left[nT\right]}_{P}=\frac{k_{nT,pro}}{div}\frac{{\left[nT\right]}_{P}}{{\left[nT\right]}_{P}+\frac{K_{nT,pro}}{div}}+Q_{nT,P,in}{\left[nT\right]}_{C}-Q_{nT,P,out}{\left[nT\right]}_{P}-k_{nT,death}{\left[nT\right]}_{P}$$

(Equation S8) $$\frac{d}{\mathrm{d}t}{\left[nT\right]}_{LN}=\frac{k_{nT,pro}}{div}\frac{{\left[nT\right]}_{LN}}{{\left[nT\right]}_{LN}+\frac{K_{nT,pro}}{div}}+Q_{nT,LN,in}{\left[nT\right]}_{C}-Q_{nT,LN,out}{\left[nT\right]}_{LN}-k_{nT,death}{\left[nT\right]}_{LN}-k_{T,act}H_{APC}H_{Ag}{\left[nT\right]}_{LN}$$

The initial amount of naïve T cells is calculated by dividing the absolute number of naïve T cells measured from healthy individuals by the T cell clonotype diversity (Autissier et al., 2010; Robins et al., 2009). *Q*_*nT*, *thym*_ represents zero-order thymic export of naïve T cells, whose rate is shown to be correlated with age by (Bains et al., 2009; Ye and Kirschner, 2002) and thus is estimated by the average ages of patients with breast cancer at diagnosis (Yeh et al., 2017). *div* represents the clonotype diversity, which is 1.11e6 and 1.16e6 for naïve CD8^+^ and CD4^+^T cell, respectively (Robins et al., 2009). Because the naïve T cell densities are sustained mainly by self-renewal in peripheral lymphoid organs, their proliferation is assumed to occur in the peripheral and the tumor-draining lymph node compartments (first terms in S7 and S8) with a rate constant, *k*_*nT*, *pro*_, estimated based on the *in vivo* measurements reported by (den Braber et al., 2012). The naïve T cell trafficking among the three compartments is adapted from the model by (Zhu et al., 1996), and the transport rates are estimated to fit naïve T cell densities at the steady state when cancer cells are not present (i.e., no naïve T cell activation by tumor antigens) to the measured naïve T cell levels in healthy individuals (Autissier et al., 2010). When cancer cells are present, Teff is activated from naïve CD8^+^T cells, and Treg and helper T cell (Th) are activated from naïve CD4^+^T cells. Figure S3 shows that the pre-treatment distribution of naïve T cells (see In silico clinical trial for definition of pre-treatment). The median densities of naïve CD4^+^ and CD8^+^T cells (6.6e5 and 4.7e5 cell/mL) are about 23% and 8% lower than those in healthy individuals (8.6e5 and 5.1e5 cell/mL), which is consistent with the clinical measurements (Autissier et al., 2010; Hueman et al., 2007).

##### T cell activation and homing

The activation of naïve T cells in the tumor-draining lymph nodes (TDLNs) depends on the number of T cells that can simultaneously interact with mature antigen-presenting cells (mAPCs) and the strength of T cell receptor (TCR)-peptide-MHC (pMHC) interactions, which are implemented as Hill functions named *H*_*APC*_ and *H*_*Ag*_, respectively (see the Antigen-presenting cell module and the Antigen module). Equations S9 and S10 describe the dynamics of proliferating T cellsupon activation, [*aT*]_*LN*_, and their final forms, [*T*]_*LN*_, (i.e., Teff, Treg, and Th).

(Equation S9) $$\frac{d}{\mathrm{d}t}{\left[aT\right]}_{LN}=k_{T,act}H_{APC}H_{Ag}{\left[nT\right]}_{LN}n_{T,clones}-\frac{k_{T,pro}}{N_{aT}}{\left[aT\right]}_{LN}$$

(Equation S10) $$\frac{d}{\mathrm{d}t}{\left[T\right]}_{LN}=\frac{k_{T,pro}}{N_{aT}}2^{N_{aT}}{\left[aT\right]}_{LN}-Q_{T,LN,out}{\left[T\right]}_{LN}-k_{T,death}{\left[T\right]}_{LN}$$

Because [*nT*]_*LN*_ represents the average level of naïve T cells of a single clonotype, the first term of Equation S9 is multiplied by the number of corresponding antigen-specific T cell clones, *n*_*T*, *clone*s_, to calculate the total number of naïve T cells that can recognize tumor neoantigens (i.e., Teff and Th) or tumor-associated self-antigens (i.e., Treg). Here, we assume that the number of neo/self-antigen-specific T cell clones equal to the number of corresponding antigen clones. For example, the number of neoantigen-specific T cell clones is estimated by the number of neoepitopes identified in TNBC (Morisaki et al., 2021; Narang et al., 2019). *k*_*T*, *act*_ is the maximal activation rate of naïve T cell by mAPCs, *k*_*T*, *pro*_ is the proliferation rate of activated T cell, and *N*_*aT*_ is the total number of divisions that an activated T cell can undergo while transitioning to its final form.

(Equation S11) $$N_{aT}=N_{TCR}+N_{costim}H_{CD28}+N_{IL2}H_{IL2}$$

In Equation S11, *N*_*TCR*_, *N*_*costim*_, and *N*_*IL*2_ represent the number of cell divisions by signals from TCR, costimuli on mAPCs, and IL-2 secreted by activated CD4^+^ helper T cells, respectively. According to the experimental data from (Marchingo et al., 2014), the effect of the three signals on activated T cell expansion can be estimated by the linear sum of the underlying signal components.

Similar to naïve T cells in the model, transport of activated T cells (i.e., Teff, Treg, and Th) is adapted from the model by (Zhu et al., 1996) and is described by Equations S12 and S13. The tumor infiltration is limited by the term $\frac{C_{total}^{2}}{C_{total}^{2}+\;K_{C,rec}}$ to constrain T cell infiltration when the number of cancer cells is low, reflecting the effect of chemoattractant (dePillis et al., 2006). This term is also used to approximate the clearance of immune and myeloid cells because of the lack of pro-inflammatory signals upon tumor eradication in Equations S14, S15, S16, S48, S49 and S50 (Kaech and Cui, 2012; Veglia et al., 2021).

(Equation S12) $$\frac{d}{\mathrm{d}t}{\left[T\right]}_{C}=Q_{T,LN,out}{\left[T\right]}_{LN}-Q_{T,P,in}{\left[T\right]}_{C}+Q_{T,P,out}{\left[T\right]}_{P}-Q_{T,T,in}{\left[T\right]}_{C}\frac{C_{total}^{2}}{C_{total}^{2}+\;K_{C,rec}}-k_{T,death}{\left[T\right]}_{C}$$

(Equation S13) $$\frac{d}{\mathrm{d}t}{\left[T\right]}_{P}=Q_{T,P,in}{\left[T\right]}_{C}-Q_{T,P,out}{\left[T\right]}_{P}-k_{T,death}{\left[T\right]}_{P}$$

Because of the differential effects of activated T cells on the tumor microenvironment, their equations are specified separately by Equations S14, S15 and S16. For tumor infiltrating Teff, additional death rates are applied to represent Teff inhibition by Treg and cancer cell, which are mediated by IL-10 and PD1-PDL1/2 interactions, respectively (Hsu et al., 2015; Wherry, 2011). For tumor infiltrating Th and Treg, differentiation of Th to Treg is incorporated, which is mediated by TGF-β and arginase-I (Batlle and Massagué, 2019; Serafini et al., 2008).

(Equation S14) $$\frac{d{\left[T_{eff}\right]}_{T}}{\mathrm{d}t}=Q_{T,T,in}{\left[T_{eff}\right]}_{C}\frac{C_{total}^{2}}{C_{total}^{2}+\;K_{C,rec}}-\left(k_{T,death}+k_{Treg}\frac{{\left[T_{reg}\right]}_{T}}{{\left[T_{eff}\right]}_{T}+{\left[T_{reg}\right]}_{T}}H_{IL10}+k_{Tcell}\frac{C_{total}}{C_{total}+{\left[T_{eff}\right]}_{T}}H_{PD1,C}+k_{cell,clear}\frac{K_{C,rec}}{C_{total}^{2}+\;K_{C,rec}}\right){\left[T_{eff}\right]}_{T}$$

(Equation S15) $$\frac{d}{\text{d}t}{\left[T_{reg}\right]}_{T}=Q_{T,T,in}{\left[T_{reg}\right]}_{C}\frac{C_{total}^{2}}{C_{total}^{2}+\;K_{C,rec}}-\left(k_{T,death}-k_{Th,Treg}H_{TGF\beta }H_{ArgI,Treg}+k_{cell,clear}\frac{K_{C,rec}}{C_{total}^{2}+\;K_{C,rec}}\right){\left[T_{reg}\right]}_{T}$$

(Equation S16) $$\frac{d}{\text{d}t}{\left[T_{h}\right]}_{T}=Q_{T,T,in}{\left[T_{h}\right]}_{C}\frac{C_{total}^{2}}{C_{total}^{2}+\;K_{C,rec}}-\left(k_{T,death}+k_{Th,Treg}H_{TGF\beta }H_{ArgI,Treg}+k_{cell,clear}\frac{K_{C,rec}}{C_{total}^{2}+\;K_{C,rec}}\right){\left[T_{h}\right]}_{T}$$

#### Antigen-presenting cell module

The APC module describes the APC recruitment into the tumor compartment, APC maturation, and mature APC transport to the tumor-draining lymph node compartment. We assume that the majority of the mAPCs come from the tumor compartment, where they uptake antigens and undergo the maturation process (Lindquist et al., 2004).

(Equation S17) $$\frac{d{\left[APC\right]}_{T}}{\mathrm{d}t}=k_{APC,death}\left(\rho_{APC}V_{T}-{\left[APC\right]}_{T}\right)-k_{APC,mat}{\left[APC\right]}_{T}H_{IL12}\left(1-H_{IL10}\right)$$

(Equation S18) $$\frac{d{\left[APC\right]}_{LN}}{\mathrm{d}t}=k_{APC,death}\left(\rho_{APC}V_{LN}-{\left[APC\right]}_{LN}\right)$$

(Equation S19) $$\frac{d{\left[mAPC\right]}_{T}}{\mathrm{d}t}=k_{APC,mat}{\left[APC\right]}_{T}H_{IL12}\left(1-H_{IL10}\right)-k_{APC,mig}{\left[mAPC\right]}_{T}-k_{mAPC,death}{\left[mAPC\right]}_{T}$$

(Equation S20) $$\frac{d{\left[mAPC\right]}_{LN}}{\mathrm{d}t}=k_{APC,mig}{\left[mAPC\right]}_{T}-k_{mAPC,death}{\left[mAPC\right]}_{LN}$$

In Equations S17, S18, S19 and S20, *k*_*APC*, *death*_ is the entry and death rate of APCs; $\rho_{APC}$ is the baseline APC density; *k*_*APC*, *mat*_ is the maturation rate that depends on concentrations of the maturation signal, IL-12, and inhibitory cytokine, IL-10 (Corinti et al., 2001); *k*_*APC*, *mig*_ is the migration rate of mAPCs from tumor to TDLNs; and *km*_*APC*, *death*_ is the death rate of mAPCs. *H*_*APC*_, which determines the rate of T cell activation in the T cell module, is calculated by Equation S21 based on the type of T cells being activated. That is, *n*_*T*, *clones*_ corresponds to the number of neoantigen clones when calculating *H*_*APC*_ for Teff and Th activation from naïve CD8^+^ and CD4^+^T cells, respectively, and it corresponds to the number of self-antigen clones when calculating *H*_*APC*_ for Treg activation from naïve CD4^+^T cells.

(Equation S21) $$H_{APC}=\frac{n_{sites,APC}{\left[mAPC\right]}_{LN}}{n_{sites,APC}{\left[mAPC\right]}_{LN}+n_{T,clones}{\left[nT\right]}_{LN}}$$

#### Antigen module

The antigen module is adapted from several well-established models to describe antigen processing and presentation by APC (Agrawal and Linderman, 1996; Chen et al., 2014b; Palsson et al., 2013). Tumor neoantigens and tumor-associated self-antigens are released upon death of cancer cells (Equation S22) and are internalized into intracellular vesicles of APC (Equation S23), where they are processed into short peptides (Equation S24). The peptides then bind with MHC molecules based on their binding affinity (Equation S25), and the pMHC complexes are presented on the cell surface to be recognized by the antigen-specific T cell receptors (Equation S26). Equations S27 and S28 describe the unbound MHC molecules in the endosome and cell surface, respectively.

(Equation S22) $$V_{T}\frac{dP_{T}}{\mathrm{d}t}=k_{dep}-k_{xP,deg}p_{T}V_{T}-k_{up}{\left[APC\right]}_{T}P_{T}V_{e}$$

(Equation S23) $$V_{e}\frac{dP_{e}}{\mathrm{d}t}=k_{up}P_{T}V_{e}-k_{P,deg}P_{e}V_{e}$$

(Equation S24) $$V_{e}\frac{dp_{e}}{\mathrm{d}t}=k_{P,deg}P_{e}V_{e}-k_{p,deg}p_{e}V_{e}-k_{P,on}M_{e}p_{e}A_{e}+k_{P,off}{\left[Mp\right]}_{e}A_{e}$$

(Equation S25) $$A_{e}\frac{d{\left[Mp\right]}_{e}}{\mathrm{d}t}=k_{P,on}M_{e}p_{e}A_{e}-k_{P,off}{\left[Mp\right]}_{e}A_{e}-k_{out}{\left[Mp\right]}_{e}A_{e}$$

(Equation S26) $$A_{s}\frac{d{\left[Mp\right]}_{s}}{\mathrm{d}t}=k_{out}{\left[Mp\right]}_{e}A_{e}-k_{P,off}{\left[Mp\right]}_{s}A_{s}$$

(Equation S27) $$A_{e}\frac{dM_{e}}{\mathrm{d}t}=k_{P,off}{\left[Mp\right]}_{e}A_{e}-k_{P,on}M_{e}p_{e}A_{e}-k_{out}M_{e}A_{e}+k_{in}M_{s}A_{s}$$

(Equation S28) $$A_{s}\frac{dM_{s}}{\mathrm{d}t}=k_{P,off}{\left[Mp\right]}_{s}A_{s}-k_{in}M_{s}A_{s}+k_{out}M_{e}A_{e}$$

Equations S22, S23, S24, S25, S26, S27 and S28 are applied for both tumor-associated self-antigens and tumor neoantigen clones. *P*_*i*_, *p*_*i*_, *M*_*i*_, and [*M*_*P*_]_*i*_ in Equations S23, S24, S25, S26, S27 and S28 represent the average concentration of the antigen, the peptide, the MHC molecule, and the pMHC complexes per APC, respectively. *V*_*i*_ and *A*_*i*_ represent the volume and the surface area. The subscripts *e* and *s* represent the APC endosomal and the surface compartment. The antigen release rate, *k*_*dep*_, is assumed equal to the rate of cancer cell death. *k*_*xP*, *dep*_ is the degradation rate of extracellular antigens. The antigen uptake rate, *k*_*up*_, antigen degradation rate, *k*_*P*, *dep*_, peptide degradation rate, *k*_*p*, *dep*_, exocytosis rate of pMHC complexes, *k*_*out*_, and internalization rate of MHC molecules, *k*_*in*_, were estimated by (Chen et al., 2014b). The peptide-MHC association rate, *k*_*P*, *on*_, was estimated by (Agrawal and Linderman, 1996), and dissociation rate, *k*_*P*, *off*_, is calculated based on the binding affinity of corresponding antigen, which can vary among different cancer types. For simplification, we assume an average binding affinity for all neoantigen/self-antigen clones to represent the overall effect of all clones.

$${\left[TCR:MHC\right]}_{tot}=\frac{1}{2}\left(\frac{{\left[Mp\right]}_{s}}{n_{T,clones}}+\;TCR_{tot}+\;K_{D,TCR}\;-\;\sqrt{{\left(\frac{{\left[Mp\right]}_{s}}{n_{T,clones}}+\;TCR_{tot}+K_{D,TCR}\right)}^{2}-4\frac{{\left[Mp\right]}_{s}}{n_{T,clones}}TCR_{tot}}\right)$$

(Equation S29) $${\left[TCR\right]}_{active}=\frac{k_{off,TCR}}{\;k_{off,TCR}+\varphi_{TCR}}{\left(\frac{k_{p,TCR}}{\;k_{p,TCR}+k_{off,TCR}}\right)}^{N_{TCR}}{\left[TCR:MHC\right]}_{tot}$$

The activation of TCR by the TCR-pMHC binding is estimated by the concentration of pMHC complex using Equation S29, where [*TCR*:*MHC*]_*tot*_ is the total number of TCR-pMHC complexes, and [*TCR*]_*active*_ is the number of active TCRs (Lever et al., 2014). Here, [*M*_*p*_]_*s*_ is divided by the number of corresponding antigen clones to represent the number of pMHC complexes of a single clonotype. This is based on the model assumption that each TCR clone can recognize one antigen clone, and each antigen clone can be recognized by one TCR clone. *TCR*_*tot*_ is the total number of TCR on T cells; $K_{D,TCR}$ is the binding affinity between TCR and pMHC complex with dissociation rate of *k*_*off*, *TCR*_; *k*_*p*, *TCR*_ is the modification rate of TCR-pMHC complexes to the signaling-competent state; $\varphi_{TCR}$ is the modification rate of TCR-pMHC complexes to the non-signaling state; and *N*_*TCR*_ is the number of modification steps. The number of active TCR determines the Hill function, *H*_*Ag*_, for naïve T cell activation in Equation S30, where *K*_*p*_ is the half-maximal active TCR level for naïve T cell activation.

(Equation S30) $$H_{Ag}=\frac{{\left[TCR\right]}_{active}}{{\left[TCR\right]}_{active}+K_{p}}\;$$

#### Pharmacokinetic (PK) module

Similarly to the methods described in our previous studies (Wang et al., 2019), the capillary permeability of antibody drugs is estimated by its Stokes-Einstein radius, which is calculated via Equation S31 (Venturoli and Rippe, 2005). Here, because atezolizumab has a molecular weight of 145 kDa, it has a Stokes-Einstein radius of 47.4 Å, by which the permeability-surface area product is estimated to be 1.5e-4 mL/(s∗100g) (Garlick and Renkin, 1970). Using a body surface area of 70 cm^2^/g, the permeability of atezolizumab between the central and the peripheral compartments is calculated to be 2e-8 cm/s, which is used as the starting value for fitting below. Because tumor blood vessels are more permeable than normal capillaries, the permeability between the central and the tumor compartments is estimated to be 3e-7 cm/s according to multiple *in vivo* studies (Thurber and Wittrup, 2012; Yuan et al., 1995); and the surface area of capillaries per tissue volume is estimated to be 28.4 cm^2^/cm^3^ for peripheral tissues and the tumor (Thurber and Wittrup, 2012). As a result, the volumetric flow rates of atezolizumab between the central and the peripheral/tumor compartments are calculated by the corresponding permeability-surface area product. The volumetric flow rate between the central and the tumor-draining lymph node compartment is estimated by an *in vivo* study (Meijer et al., 2017).

(Equation S31) $${\text{a}}_{\text{e}}=0.483*{\left(MW\right)}^{0.386}$$

Based on the parameter estimation above, we further fit the volumetric flow rate between the central and the peripheral compartments (*Q*_*P*_), the clearance rate (*k*_*cl*_), and the volume fractions of plasma (in the central compartment) (*γ*_*C*_) and the interstitial space available to atezolizumab (in the peripheral compartment) (*γ*_*P*_) to match its clinically measured plasma concentration (Stroh et al., 2017). Figure S4 shows the model predicted plasma concentration of atezolizumab with the clinically measured values.

(Equation S32) $$V_{C}\frac{d{\left[A\right]}_{C}}{\mathrm{d}t}=Q_{P}\left(\frac{{\left[A\right]}_{P}}{\gamma_{P}}-\frac{{\left[A\right]}_{C}}{\gamma_{C}}\right)+Q_{LN}\left(\frac{{\left[A\right]}_{LN}}{\gamma_{LN}}-\frac{{\left[A\right]}_{C}}{\gamma_{C}}\right)+Q_{T}\left(\frac{{\left[A\right]}_{T}}{\gamma_{T}}-\frac{{\left[A\right]}_{C}}{\gamma_{C}}\right)+Q_{LD}\frac{{\left[A\right]}_{LN}}{\gamma_{LN}}-k_{cl}{\left[A\right]}_{C}$$

(Equation S33) $$V_{P}\frac{d{\left[A\right]}_{P}}{\mathrm{d}t}=-Q_{P}\left(\frac{{\left[A\right]}_{P}}{\gamma_{P}}-\frac{{\left[A\right]}_{C}}{\gamma_{C}}\right)$$

(Equation S34) $$V_{T}\frac{d{\left[A\right]}_{T}}{\mathrm{d}t}=-Q_{T}\left(\frac{{\left[A\right]}_{T}}{\gamma_{T}}-\frac{{\left[A\right]}_{C}}{\gamma_{C}}\right)-Q_{LD}\frac{{\left[A\right]}_{T}}{\gamma_{T}}$$

(Equation S35) $$V_{LN}\frac{d{\left[A\right]}_{LN}}{\text{d}t}=-Q_{LN}\left(\frac{{\left[A\right]}_{LN}}{\gamma_{LN}}-\frac{{\left[A\right]}_{C}}{\gamma_{C}}\right)+Q_{LD}\frac{{\left[A\right]}_{T}}{\gamma_{T}}-Q_{LD}\frac{{\left[A\right]}_{LN}}{\gamma_{LN}}$$

In Equations S32, S33, S34 and S35, [*A*]_*i*_ is the antibody concentration, *V*_*i*_ is the compartment volume, *Q*_*i*_ is the volumetric flow rate between the central and the corresponding compartment, *γ*_*i*_ is volume fraction of interstitial space available to the antibody, *k*_*cl*_ is clearance rate, and *Q*_*LD*_ is the rate of lymphatic drainage from tumor to TDLNs and from TDLNs to plasma (Zhu et al., 1996). Subscripts *C, P, LN, T* represent the central, peripheral, tumor-draining lymph node, and tumor compartments, respectively.

#### Checkpoint module

##### Dynamics of PD-1-related checkpoint molecules

Interactions among PD-1, PD-L1, CD80, and anti-PD-(L)1 antibodies are adapted from our previously published model (Jafarnejad et al., 2019), which are integrated into a sub-compartment representing the immunological synapse in the present model. We assume that the ligands and receptors are evenly distributed on the cell surface so that their densities in the synapse are calculated by dividing their expression levels by the total cell surface area. We also assume that the explicit representation of the diffusive entry of surface molecules to the synapse is negligible because of its rapid dynamics. Instead, the area of the synapse is increased by a factor of 3 to account for the effect of diffusion (Jansson et al., 2005). The numbers of checkpoint molecules on cell surface are estimated based on measurements using quantitative flow cytometry with fluorescent beads (Cheng et al., 2013), which are then scaled up to account for possible underestimation of PD-L1/2 level by QuantiBRITE bead measurements (Mkrtichyan et al., 2012). These parameters are varied in a wide range in the virtual patient generation to account for the uncertainty and inter-individual variability.

(Equation S36) $$\frac{d\left[PD1:PDL1\right]}{\mathrm{d}t}=\frac{k_{on,PD1,PDL1}}{d_{syn}}\left[PD1\right]\left[PDL1\right]-k_{off,PD1,PDL1}\left[PD1:PDL1\right]$$

(Equation S37) $$\frac{d\left[PD1:PDL2\right]}{\mathrm{d}t}=\frac{k_{on,PD1,PDL2}}{d_{syn}}\left[PD1\right]\left[PDL2\right]-k_{off,PD1,PDL2}\left[PD1:PDL2\right]$$

(Equation S38) $$\frac{d\left[PDL1:aPDL1\right]}{\mathrm{d}t}=2k_{on,PDL1,aPDL1}\left[PDL1\right]\frac{{\left[aPDL1\right]}_{T}}{\gamma_{T}}-k_{off,PDL1,aPDL1}\left[PDL1:aPDL1\right]-\chi_{aPDL1}\frac{k_{on,PDL1,aPDL1}}{d_{syn}N_{A}}\left[PDL1:aPDL1\right]\left[PDL1\right]+2k_{off,PDL1,aPDL1}\left[PDL1:aPDL1:PDL1\right]$$

(Equation S39) $$\frac{d\left[PDL1:aPDL1:PDL1\right]}{\mathrm{d}t}=\chi_{aPDL1}\frac{k_{on,PDL1,aPDL1}}{d_{syn}N_{A}}\left[PDL1:aPDL1\right]\left[PDL1\right]-2k_{off,PDL1,aPDL1}\left[PDL1:aPDL1:PDL1\right]$$

(Equation S40) $$\frac{d\left[PDL1\right]}{\mathrm{d}t}=\frac{k_{out,PDL1}}{A_{syn}}\frac{\left[IFN\gamma \right]}{\left[IFN\gamma \right]+IFN\gamma_{50}}\left(1-\frac{{\left[PDL1\right]}_{total}}{r_{PDL1,IFN\gamma }{\left[PDL1\right]}_{baseline}}\right)+k_{in,PDL1}\left({\left[PDL1\right]}_{baseline}-{\left[PDL1\right]}_{total}\right)-\frac{k_{on,PD1,PDL1}}{d_{syn}}\left[PD1\right]\left[PDL1\right]+k_{off,PD1,PDL1}\left[PD1:PDL1\right]-2k_{on,PDL1,aPDL1}\left[PDL1\right]\frac{{\left[aPDL1\right]}_{T}}{\gamma_{T}}+k_{off,PDL1,aPDL1}\left[PDL1:aPDL1\right]-\chi_{aPDL1}\frac{k_{on,PDL1,aPDL1}}{d_{syn}N_{A}}\left[PDL1:aPDL1\right]\left[PDL1\right]+2k_{off,PDL1,aPDL1}\left[PDL1:aPDL1:PDL1\right]-2\frac{k_{on,CD80,PDL1}}{d_{syn}}\left[PDL1\right]\left[CD80\right]+2k_{off,CD80,PDL1}\left[PDL1:CD80\right]$$

(Equation S41) $$\frac{d\left[PDL2\right]}{\mathrm{d}t}=r_{PDL2}\frac{k_{out,PDL1}}{A_{syn}}\frac{\left[IFN\gamma \right]}{\left[IFN\gamma \right]+IFN\gamma_{50}}\left(1-\frac{{\left[PDL2\right]}_{total}}{r_{PDL1,IFN\gamma }{\left[PDL1\right]}_{baseline}r_{PDL2}}\right)+k_{in,PDL1}\left({\left[PDL1\right]}_{baseline}r_{PDL2}-{\left[PDL2\right]}_{total}\right)-\frac{k_{on,PD1,PDL2}}{d_{syn}}\left[PD1\right]\left[PDL2\right]+k_{off,PD1,PDL2}\left[PD1:PDL2\right]$$

Equations S36, S37, S38, S39, S40 and S41 describe the PD-1-related dynamics in the model. The model species represent the 2-D densities of the checkpoint molecules in the synapse with a surface area, *A*_*syn*_. *k*_*on*_ and *k*_*off*_are the association and dissociation rates of checkpoint interactions; the coefficients for *k*_*on*_ and *k*_*off*_ of antibody-target binding in Equation S38 represent the stochiometric corrections because of antibody bivalency (Harms et al., 2014); *γ*_*T*_ is the volume fraction of tumor interstitium that is available to the antibody; $\chi_{aPDL1}$ is the intrinsic antibody cross-arm binding efficiency (Harms et al., 2014); the denominator, *d*_*syn*_, is the thickness of the confinement space between two cells during the interaction, which aims to transfer the 3-D binding affinity to 2-D (Jansson et al., 2005); *N*_*A*_ is Avogadro’s number. *k*_*out*, *PDL1*_is the expression rate of PD-L1/2 by IFNγ; *r*_*PDL*1, *IFNγ*_ is the number of fold increase of PD-L1/2 expression from baseline level by IFNγ; *k*_*in*, *PDL1*_ is the degradation/internalization rate of unbound PD-L1/2 molecules (Shin et al., 2017); *r*_*PDL*2_ is the ratio of PDL2 to PDL1. The number of bound PD-1 molecules to PD-L1/2 molecules in the tumor determines the Hill function, *H*_*PD*_1, for inhibitory effects of Treg and cancer cell on Teff (Equation S42).

(Equation S42) $$H_{PD1}=\frac{{\left(\left[PD1:PDL1\right]+\left[PD1:PDL2\right]\right)}^{n_{PD1}}}{{\left(\left[PD1:PDL1\right]+\left[PD1:PDL2\right]\right)}^{n_{PD1}}+PD1_{50}^{n_{PD1}}\;}$$

##### Dynamics of CTLA-4-related checkpoint molecules

Interactions among CTLA-4, CD28, CD80/86, and anti-CTLA-4 antibody are modeled similarly based on a published model (Jansson et al., 2005). Briefly, CD28 and CTLA-4 expressed on naïve T cells compete for CD80 and CD86 on APCs. CD28 and CD86 are monovalent, whereas CD80 and CTLA-4 are bivalent. Therefore, CD28 and CD86 can form a monovalent complex, whereas CD28 and CD80, CD86 and CTLA-4 can form bivalent complexes via *trans* interactions; Also, because of the bivalency of CD80 and CTLA-4, they can form three types of multivalent complexes in a zipper-like fashion (Jansson et al., 2005). In addition, PD-L1 can disrupt CD80 homodimers and form PDL1:CD80 heterodimers via *cis* interactions, which allows CD80 to bind with CD28 but potentially weaken interactions between CD80 and CTLA-4 (Equations S43, S44, S45 and S46) (Zhao et al., 2019b). CD28 is a co-stimulatory signal that enhances the naïve T cell activation by increasing the number of T cell divisions. Because CTLA-4 outcompetes CD28 because of its higher binding affinity to CD80/CD86, the blockade of CTLA-4 restores ligand availability for CD28 that leads to enhanced T cell activation and proliferation. Similar to those in the PD-1-related dynamics, stoichiometric corrections are incorporated for antibody bivalency and dimerization of CTLA-4 and CD86 on cell surface which also results in bivalency (Bhatia et al., 2005; Harms et al., 2014; Linsley et al., 1995). The ratio of bound CD28 molecules to CD80/86 molecules on mAPCs determines the Hill function, *H*_*CD*28_, to calculate the number of T cell divisions by costimulatory signals (Equations S47 and S48).

(Equation S43) $$\frac{d\left[CD80m\right]}{\mathrm{d}t}=-\frac{2k_{on,CD80:CD80}}{d_{syn}}\left[CD80m\right]\left[CD80m\right]+k_{off,CD80:CD80}\left[CD80\right]-\frac{k_{on,CD80:PDL1}}{d_{syn}}\left[CD80m\right]\left[PDL1\right]+k_{off,CD80:PDL1}\left[PDL1:CD80\right]$$

(Equation S44) $$\frac{d\left[PDL1:CD80\right]}{\mathrm{d}t}=\frac{k_{on,CD80,PDL1}}{d_{syn}}\left[CD80m\right]\left[PDL1\right]-k_{off,CD80,PDL1}\left[PDL1:CD80\right]-\frac{k_{on,CD28,CD80}}{d_{syn}}\;\left[PDL1:CD80\right]\left[CD28\right]+k_{off,CD28,CD80}\left[PDL1:CD80:CD28\right]-\frac{k_{on,CTLA4,CD80}}{d_{syn}}\left[PDL1:CD80\right]\left[CTLA4\right]+k_{off,CTLA4,CD80}\left[PDL1:CD80:CTLA4\right]$$

(Equation S45) $$\frac{d\left[PDL1:CD80:CD28\right]}{\mathrm{d}t}=\frac{k_{on,CD28,CD80}}{d_{syn}}\left[PDL1:CD80\right]\left[CD28\right]-k_{off,CD28,CD80}\left[PDL1:CD80:CD28\right]$$

(Equation S46) $$\frac{d\left[PDL1:CD80:CTLA4\right]}{\mathrm{d}t}=\frac{k_{on,CTLA4,CD80}}{d_{syn}}\left[PDL1:CD80\right]\left[CTLA4\right]-k_{off,CTLA4,CD80}\left[PDL1:CD80:CTLA4\right]$$

(Equation S47) $$CD28_{bound}=\left[CD28:CD80\right]+\left[CD28:CD86\right]+2*\left[CD28:CD80:CD28\right]+\left[PDL1:CD80:CD28\right]$$

(Equation S48) $$H_{CD28}=\frac{{\left(CD28_{bound}\right)}^{n_{CD28}}}{{\left(CD28_{bound}\right)}^{n_{CD28}}+CD28_{50}^{n_{CD28}}\;}$$

Overall, the density of checkpoint molecules in the model represents their average expression level on all cancer/immune cells in the tumor, so that the calculated Hill functions represent the overall effect mediated by PD-1 and CD28.

#### Myeloid-derived suppressor cells (MDSC) module

MDSC module describes MDSC recruitment into the tumor and secretion of arginase-I (Arg-I) and nitric oxide (NO) with their inhibitory effects on T cells. Equation S49 describes MDSC recruitment mediated by CCL2, which is secreted by cancer cells. *k*_*MDSC*, *mig*_ represents the recruitment rate estimated by the median MDSC density in patients with breast cancer (Diaz-Montero et al., 2009).

(Equation S49) $$\frac{d\left[MDSC\right]}{\mathrm{d}t}=k_{MDSC,mig}V_{T}H_{CCL2}-\left(k_{MDSC,death}+k_{cell,clear}\frac{K_{C,rec}}{C_{total}^{2}+\;K_{C,rec}}\right)\left[MDSC\right]$$

The major immunosuppressive factors secreted by MDSC are assumed to be Arg-I and NO, whose expression rates are estimated based on *in vitro* experiments on breast cancer cells (Serafini et al., 2008). Because only the enzymatic activity of Arg-I is measured in enzyme unit, mU, we use mU as a placeholder of Arg-I concentration in the model, assuming that the protein concentration is proportional to the enzymatic activity. The unit of its production rate is then set to be mU∗(microliter)/cell/day to estimate the amount of Arg-I produced by MDSC per day. The units of production rates of NO and CCL2 are set to be nanomole/cell/day. Although both Arg-I and NO inhibit cytotoxic killing of cancer cells by Teff, only Arg-I facilitates Treg expansion in the tumor (Serafini et al., 2008).

#### Nab-paclitaxel module

##### Pharmacokinetics

The plasma concentration of nab-paclitaxel is simulated by integrating a published 3-compartment PK model, which was calibrated by clinical measurements from eight clinical trials involving patients with advanced or metastatic solid tumors (Chen et al., 2014a). The intratumoral concentration of nab-paclitaxel is then approximated by tumor-to-plasma ratio measured by *in vivo* studies (Rajeshkumar et al., 2016; Yang et al., 2007). The PK parameters and body surface area are varied within the clinically reported 95% confidence intervals to represent the inter-individual variability (Chen et al., 2014a). Tumor-to-plasma ratio of nab-paclitaxel is also varied during virtual patient generation (Table S1).

##### Pharmacodynamics

Although the cytotoxic effect of nab-paclitaxel on cancer cells is well-established by *in vitro* and *in vivo* data from breast cancer mouse models, one of the challenges in chemotherapy is the development of chemo-resistance, which can be characterized into two types: intrinsic and acquired (Ji et al., 2019). Intrinsic chemo-resistance is caused by pre-existing mechanisms before the therapy begins, whereas the acquired chemo-resistance appears during the therapy. As a result, the effective concentration (EC50) of nab-paclitaxel can be different among the patients and change over time. To account for the intrinsic chemo-resistance, different values of EC50 are assigned for nab-paclitaxel on the initial cancer clone to each virtual patient. The distribution of EC50 is estimated by data reported in the Genomics of Drug Sensitivity in Cancer (GDSC) database (Yang et al., 2013). Further, we implement an additional cancer clone (*C*_2_) using the cancer module, which represents the chemo-resistant cancer cells induced during treatments. We assume that the chemo-resistant clone has the same growth dynamics as the initial cancer clone (*C*_1_) but with a 100 times higher EC50 of nab-paclitaxel (Němcová-Fürstová et al., 2016). The development of the chemo-resistant clone from the initial cancer clone is simplified by a first-order reaction mediated by TGF-β (Bhola et al., 2013), with a reaction rate of *k*_*C*, *resist*_C_1_H_*TGFβ*_. In addition to its cytotoxic activity, nab-paclitaxel is reported to induce VEGF-A expression with low doses (Volk et al., 2008) and apoptosis of endothelial cell with a dose-dependent rate (Mollard et al., 2017). The rates of the drug-induced effects are estimated by preclinical studies on breast cancer (Table S2).

#### Macrophage module

The Macrophage module adds M1- and M2-like macrophages, IL-10, and IL-12 into the QSP model as new model species. We assume that pro-inflammatory M1-like macrophages are first recruited into the tumor by CCL2 (Opalek et al., 2007), where they undergo reversible macrophage polarization. M2 polarization is mediated by TGF-β and IL-10 (Makita et al., 2015; Zhang et al., 2016), and M1 polarization is mediated by IFNγ and IL-12 (Martinez and Gordon, 2014; Watkins et al., 2007). One of the major functions of M1-like macrophage is phagocytosis of cancer cells, which is described in Equation S1. This process is known to be inhibited by IL-10 and checkpoints molecules including SIRPα and PD-1 expressed on macrophages (Bian et al., 2016; Gordon et al., 2017). Other effects of macrophages are mediated by cytokines. Specifically, IL-12 is secreted by M1-like macrophages (Dorman and Holland, 2000); IL-10, TGF-β, and VEGF-A are secreted by M2-like macrophages (Martinez and Gordon, 2014). Functions of these cytokines are demonstrated in various equations above. For both newly added and pre-existing cytokines in the model, secretion rates are calibrated by the cytokine concentration in biopsy samples from breast cancer. If biopsy samples are not available, we use the serum concentration measured from patients with breast cancer.

(Equation S50) $$\frac{d}{\mathrm{d}t}{\left[Mac\right]}_{M1}=k_{Mac,mig}V_{T}H_{CCL2}+k_{M1,pol}\left(H_{IL12}+H_{IFN\gamma }\right){\left[Mac\right]}_{M2}-\left(k_{M2,pol}\left(H_{TGF\beta }+H_{IL10}\right)+k_{Mac,death}+k_{cell,clear}\frac{K_{C,rec}}{C_{total}^{2}+\;K_{C,rec}}\right){\left[Mac\right]}_{M1}$$

(Equation S51) $$\frac{d}{\mathrm{d}t}{\left[Mac\right]}_{M2}=k_{M2,pol}\left(H_{TGF\beta }+H_{IL10}\right){\left[Mac\right]}_{M1}-\left(k_{M1,pol}\left(H_{IL12}+H_{IFN\gamma }\right)+k_{Mac,death}+k_{cell,clear}\frac{K_{C,rec}}{C_{total}^{2}+\;K_{C,rec}}\right){\left[Mac\right]}_{M2}$$

(Equation S52) $$\frac{d\left[CD47:SIRP\alpha \right]}{\mathrm{d}t}=\frac{k_{on,CD47:SIRP\alpha }}{d_{syn}}\left[CD47\right]\left[SIRP\alpha \right]-k_{off,CD47:SIRP\alpha }\left[CD47:SIRP\alpha \right]$$

(Equation S53) $$H_{SIRP\alpha }=\frac{{\left[CD47:SIRP\alpha \right]}^{n_{SIRP\alpha }}\;}{{\left[CD47:SIRP\alpha \right]}^{n_{SIRP\alpha }}+SIRP\alpha_{50}^{n_{SIRP\alpha }}}$$

(Equation S54) $$H_{Mac,C}=1-\left(1-H_{SIPR\alpha }\right)\left(1-H_{PD1,M}\right)$$

Equations S50 and S51 describe the dynamics of macrophages. *k*_*Mac*, *mig*_ is the recruitment rate; *k*_*M*1, *pol*_ and *k*_*M*2, *pol*_ are rates of M1 and M2 polarization; *k*_*Mac*, *death*_ is the death rate of macrophage; and *k*_*cell*, *clear*_ is the clearance rate of macrophage upon tumor eradication (see T cell activation and homing). Checkpoint interaction between CD47 and SIRPα is described by Equation S52 similarly to the interactions in *Checkpoint module*. Equations S42,S53 and S54 calculate the Hill function, *H*_*Mac*, *C*_, which represents the overall inhibitory effect of immune checkpoint on phagocytosis by TAMs (Equation S1).

#### In silico clinical trial

According to the distribution estimated for parameters of interest (Table S1), a virtual patient population is generated using Latin-Hypercube Sampling (LHS). Each randomly generated parameter set, which represents a patient in principle, is substituted into the model for simulation. Simulations are performed in MATLAB SimBiology Toolbox (MathWorks, Natick, MA) using sundials solver with absolute and relative tolerance of 1e-9 and 1e-6, respectively. For each virtual patient, the randomly generated initial tumor diameter is inputted to represent their pre-treatment tumor size, and the model is simulated starting from a small number of cancer cells until the tumor reaches the desired pre-treatment tumor size. Virtual patients that do not reach the desired pre-treatment tumor size are eliminated from the simulation. Once tumor reaches the desired pre-treatment size, drug administration is simulated via a SimBiology dose object, which specifies the dose amount, the infusion time, and the dose schedule; and tumor growth is then simulated for 400 days. At the post-processing step, the virtual patients are further filtered to make sure that their characteristics (e.g., T cell and macrophage densities) fall within physiologically reasonable ranges. Eventually, tumor diameter and percentage changes from baseline are calculated assuming a spherical tumor by Equations S55 and S56. Here, *D*_*T*_ is the tumor diameter, *D*_*T*, *perc*_ is the percentage change from baseline, and $D_{T}^{0}$ is the initial tumor diameter. The response status of each virtual patient is then evaluated by *D*_*T*, *perc*_ based on RECIST 1.1 (Eisenhauer et al., 2009) or immune-related response criteria (irRC) (Wolchok et al., 2009). Duration of response is calculated from the first time point when partial or complete response is observed until progressive disease (as defined by RECIST 1.1) or the end of the simulation.

(Equation S55) $$D_{T}=2{\left(\frac{3}{4\pi }V_{T}\right)}^{\frac{1}{3}}$$

(Equation S56) $$D_{T,perc}\;=\left(\frac{D_{T}\;-\;D_{T}^{0}}{D_{T}^{0}}\right)*100\%$$

### Quantification and statistical analysis

Global uncertainty and sensitivity analysis was performed by Latin-Hypercube Sampling and Partial Rank Correlation Coefficient (LHS-PRCC) methods (Marino et al., 2008) to examine the impact of varied parameters on model observations. For comparison between model predictions and clinical observations, bootstrap sampling is used to resample the virtual patient population with a sample size matching the trial size (i.e., the number of patients enrolled in the trial). The bootstrap median and the 95 percentile confidence intervals are then calculated. Statistical analyses were performed in MATLAB 2020b (MathWorks, Natick, MA) and RStudio 1.4 (PBC, Boston, MA).

## Data Availability

• All original code has been deposited at Zenodo and is publicly available as of the date of publication. DOIs are listed in the key resources table. • Any additional information required to reanalyze the data reported in this paper is available from the lead contacton request.

## Supplemental information

Document S1. Figures S1–S4

Table S1. Parameter distribution and range used in virtual patient generation, related to STAR Methods

Table S2. List of model parameters with name, value, unit, and description, related to STAR Methods

Table S3. List of model species with initial condition and description, related to STAR Methods

Table S4. List of model reactions with reaction rate and description, related to STAR Methods

Table S5. List of model rules, related to STAR Methods

Table S6. List of model compartments with name, capacity, unit, and description, related to STAR Methods
