# Supplementary material for: Dynamics of tumor-associated macrophages in a quantitative systems pharmacology model of immunotherapy in triple-negative breast cancer
Source: iScience. 2022 Jun 30;25(8):104702. doi: 10.1016/j.isci.2022.104702 (PMC9287616; doi:10.1016/j.isci.2022.104702)
Supplement: Document S1. Figures S1–S4 [file mmc1.pdf]

iScience, Volume *25*

Supplemental information

Dynamics of tumor-associated macrophages in a quantitative systems pharmacology model of immunotherapy in triple-negative breast cancer

Hanwen Wang, Chen Zhao, Cesar A. Santa-Maria, Leisha A. Emens, and Aleksander S. Popel

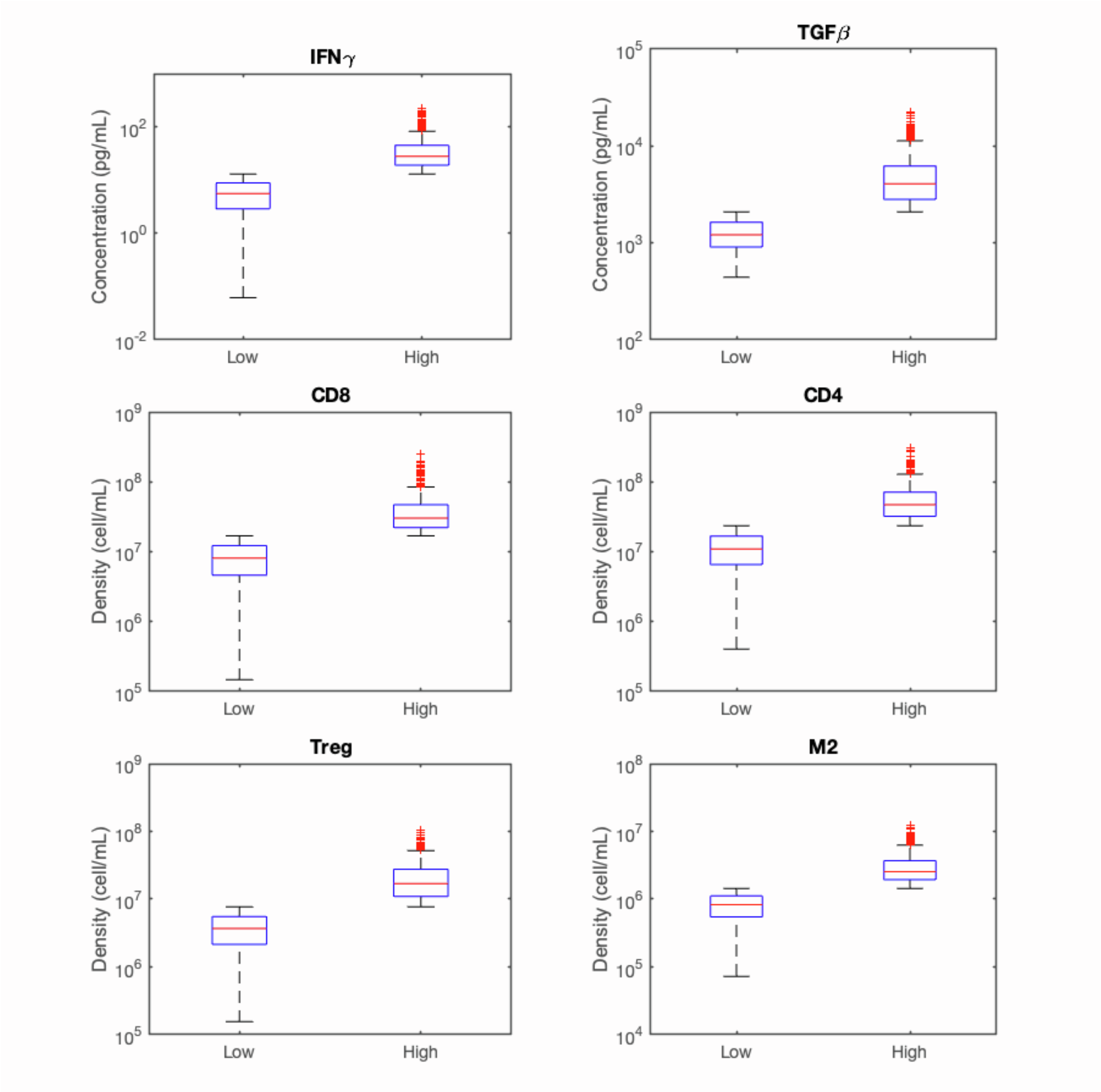

Figure S1. Distribution of cellular density and cytokine concentration in virtual patient subgroups divided by the median density of M1-like macrophages. Related to Figure 6.

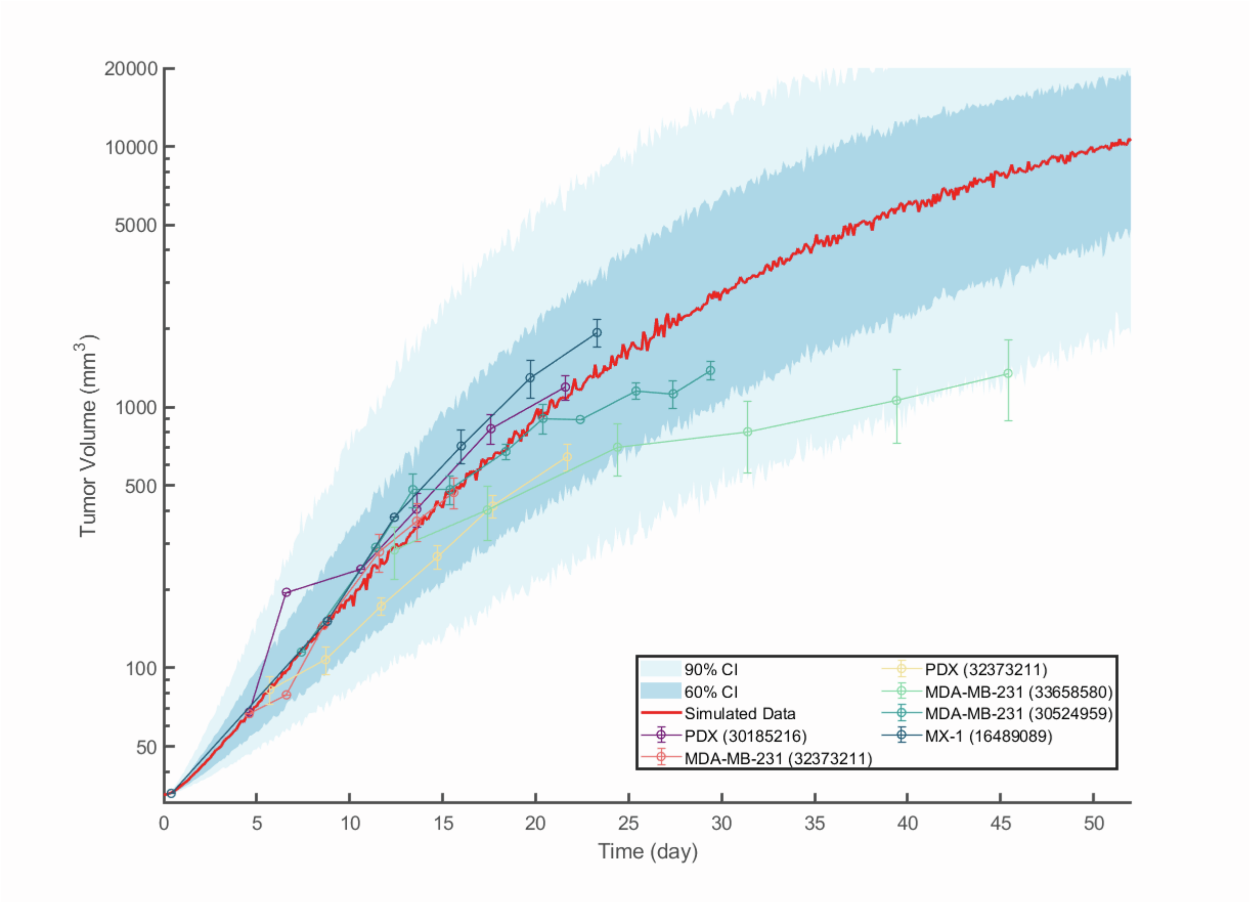

Figure S2. Time-dependent tumor volume profile. Mean tumor volumes with standard errors from preclinical studies using various types of TNBC xenograft mouse models are plotted. PMID of each study is included in the legend. Red line represents bootstrap median of the simulated tumor growth, with 60% and 90% bootstrap confidence intervals. PDX, patient-derived xenograft. Related to STAR Methods.

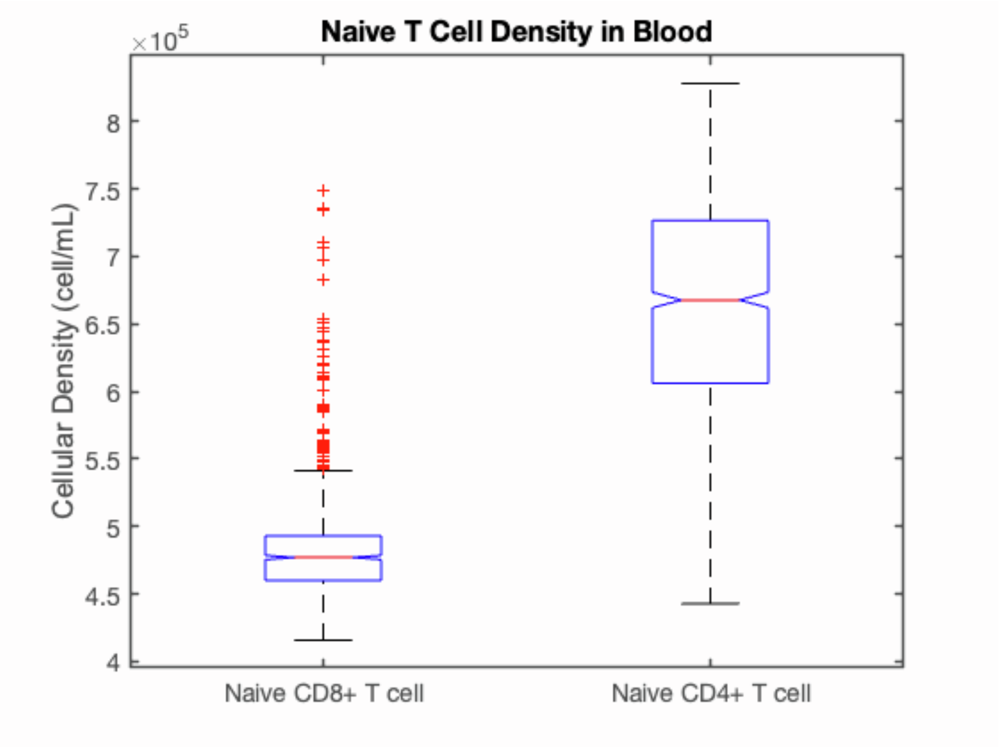

Figure S3. Pre-treatment density naïve T cells in virtual patient population. The median densities of naïve $CD4^+$ and $CD8^+$ T cells in the blood are 6.6e5 and 4.7e5 cell/mL, respectively. Related to STAR Methods.

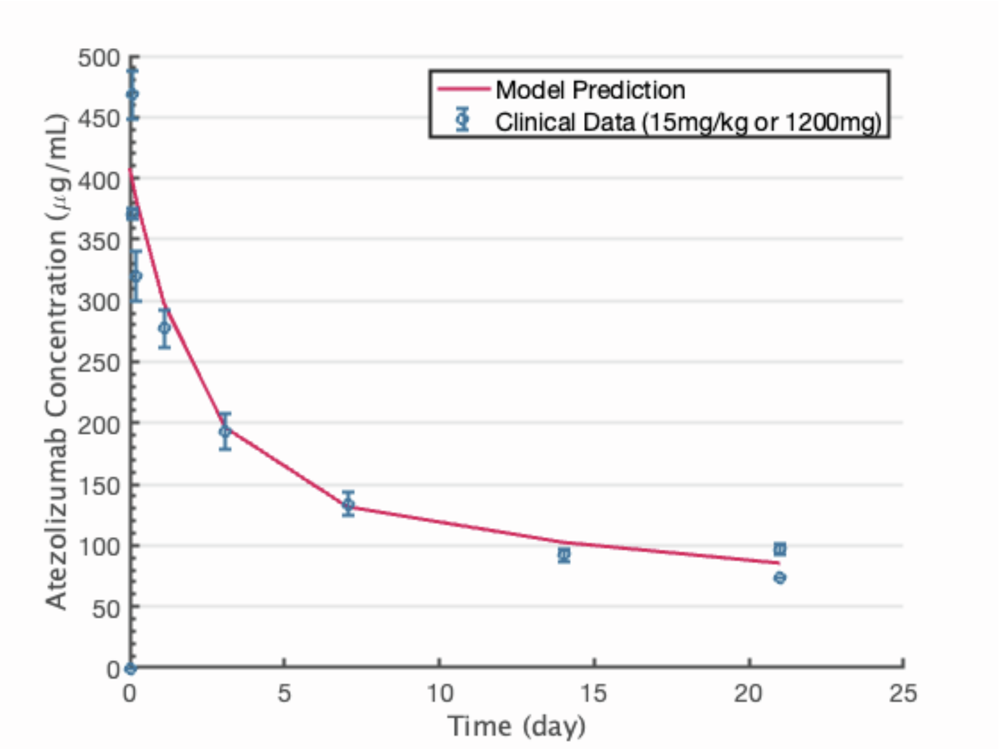

Figure S4. Plasma concentration of atezolizumab. Point data represent the plasma concentration measured after administration of 15 mg/kg or 1200 mg atezolizumab (Stroh et al., 2017). Related to STAR Methods.
